# How weaponizing disinformation can bring down a city’s power grid

**DOI:** 10.1371/journal.pone.0236517

**Published:** 2020-08-12

**Authors:** Gururaghav Raman, Bedoor AlShebli, Marcin Waniek, Talal Rahwan, Jimmy Chih-Hsien Peng

**Affiliations:** 1 Department of Electrical and Computer Engineering, National University of Singapore, Singapore; 2 Computer Science, New York University, Abu Dhabi, United Arab Emirates; Xidian University, CHINA

## Abstract

Social media has made it possible to manipulate the masses via disinformation and fake news at an unprecedented scale. This is particularly alarming from a security perspective, as humans have proven to be one of the weakest links when protecting critical infrastructure in general, and the power grid in particular. Here, we consider an attack in which an adversary attempts to manipulate the behavior of energy consumers by sending fake discount notifications encouraging them to shift their consumption into the peak-demand period. Using Greater London as a case study, we show that such disinformation can indeed lead to unwitting consumers synchronizing their energy-usage patterns, and result in blackouts on a city-scale if the grid is heavily loaded. We then conduct surveys to assess the propensity of people to follow-through on such notifications and forward them to their friends. This allows us to model how the disinformation may propagate through social networks, potentially amplifying the attack impact. These findings demonstrate that in an era when disinformation can be weaponized, system vulnerabilities arise not only from the hardware and software of critical infrastructure, but also from the behavior of the consumers.

## Introduction

Social media has dramatically altered the ways in which conflicts are fought. By allowing belligerents to command the public narrative, these technologies have created a paradigm wherein the most viral information can influence the outcome of wars [1]. This phenomenon has been exacerbated by social media algorithms that value virality over veracity [2, 3]. Unsurprisingly, many notable skirmishes in recent years have used disinformation to manipulate peoples’ behavior [1, 4]. Such campaigns have become particularly effective due to the ever-increasing prevalence of big data and machine learning techniques that allow the behavioral patterns of the masses to be analyzed with unprecedented precision. Among the clearest manifestations of such campaigns are the alleged Russian interference into the 2016 US presidential election and the Brexit referendum [5, 6]. These incidents suggest that the microtargeting capabilities provided by companies such as Cambridge Analytica [7] can be *weaponized* [8] to influence the long-term decisions of a society. While many studies have analyzed campaigns targeting long-term social behavior manipulation [2, 9–12], little attention has been given to targeted attacks that use disinformation as a weapon to manipulate social behavior within a limited time span.

One particularly sensitive target that is vulnerable to behavioral manipulation is critical infrastructure, the attack of which may have drastic implications nationwide. For instance, despite high levels of security, human operators proved to be the weakest link during the Stuxnet attack on the Iranian nuclear program, unwittingly introducing malware into the facilities [13, 14]. Another attack of this kind that drew concern from governments worldwide was the Ukrainian power grid cyberattack of 2015 [15, 16]. In this incident, attackers deliberately cut off the power supply for 230,000 residents for several hours using operator credentials harvested through one particular form of disinformation, namely, spear-phishing [17].

In this study, we focus on the power grid—a choice motivated by the devastation caused by historical power outages including human casualties and massive financial losses [18–20]. Yet, while numerous blackout prevention and mitigation strategies have been proposed in the literature [21–32], the link between disinformation and blackouts has never been studied to date. Driven by this observation, we seek to answer the following question: can an adversary bring down a city’s power grid using disinformation without any physical or cyber intrusions? The main contribution of this analysis is to assess whether an adversary could attack the power distribution system not by targeting its hardware or software infrastructure, but by focusing entirely on manipulating individual consumers’ behavior.

The rest of the paper is organized as follows. We begin by describing the mechanism of a disinformation attack on the power grid, and then evaluate the impact of such an attack considering the distribution network of the Greater London area as a case study. Subsequently, to quantify the risk posed by disinformation attacks, we perform analyses to estimate what disinformation follow-through rates could be achieved by an adversary in reality. We conclude by highlighting the implications of our study.

## Attack impact on the power grid

**Fig 1:**
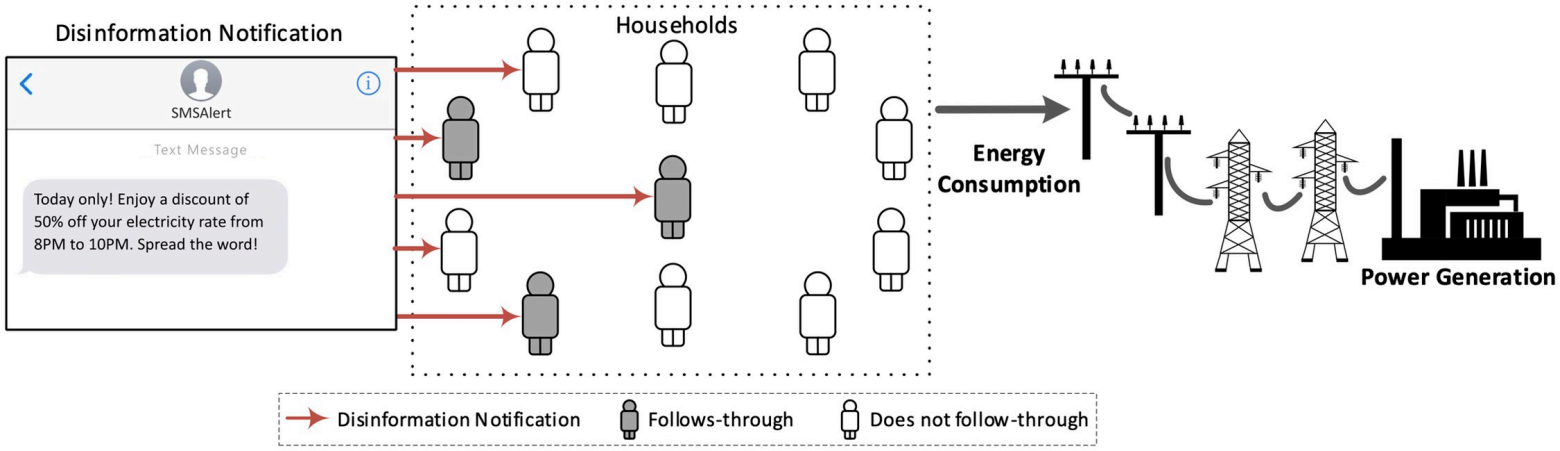
An overview of a disinformation attack on the power system. Illustrating how the disinformation attack is launched from an attacker, thereby altering the energy consumption patterns of a portion of the population. Importantly, not every recipient follows-through on the notification.

We consider an attack in which an adversary attempts to manipulate the behavior of citizens by sending fake discount notifications encouraging them to shift their energy consumption into the peak-demand period. Such a shift may result in the tripping of overloaded power lines, leading to blackouts (see Methods). An overview of this attack and the disinformation message are shown in Fig 1. Ultimately, the success of such an attack depends on the *follow-through rate*, i.e., the fraction of people who behave as intended by the attacker. We analyze the impact caused by such behavioral manipulation on the power grid. To this end, we modeled the power grid of Greater London (see S1 Note in S1 File) and simulated the behavior of residential energy consumers. Importantly, our model considers residential electric vehicle (EV) adoption since the owners of such EVs control a substantial amount of deferrable energy, and thus can cause greater harm when manipulated by an adversary. We vary the EV adoption level in the city, and model the capacity upgrades that are necessary for the grid to support the demand corresponding to each such level [33–35]; see Methods. Note that although the EV charging demand is only one component of the total deferrable demand, it nevertheless accounts for a significant part of the latter. Therefore, in the following analysis, we use increasing EV adop-tion level as a synecdoche for the increasing amount of deferrable demand in the grid.

**Fig 2:**
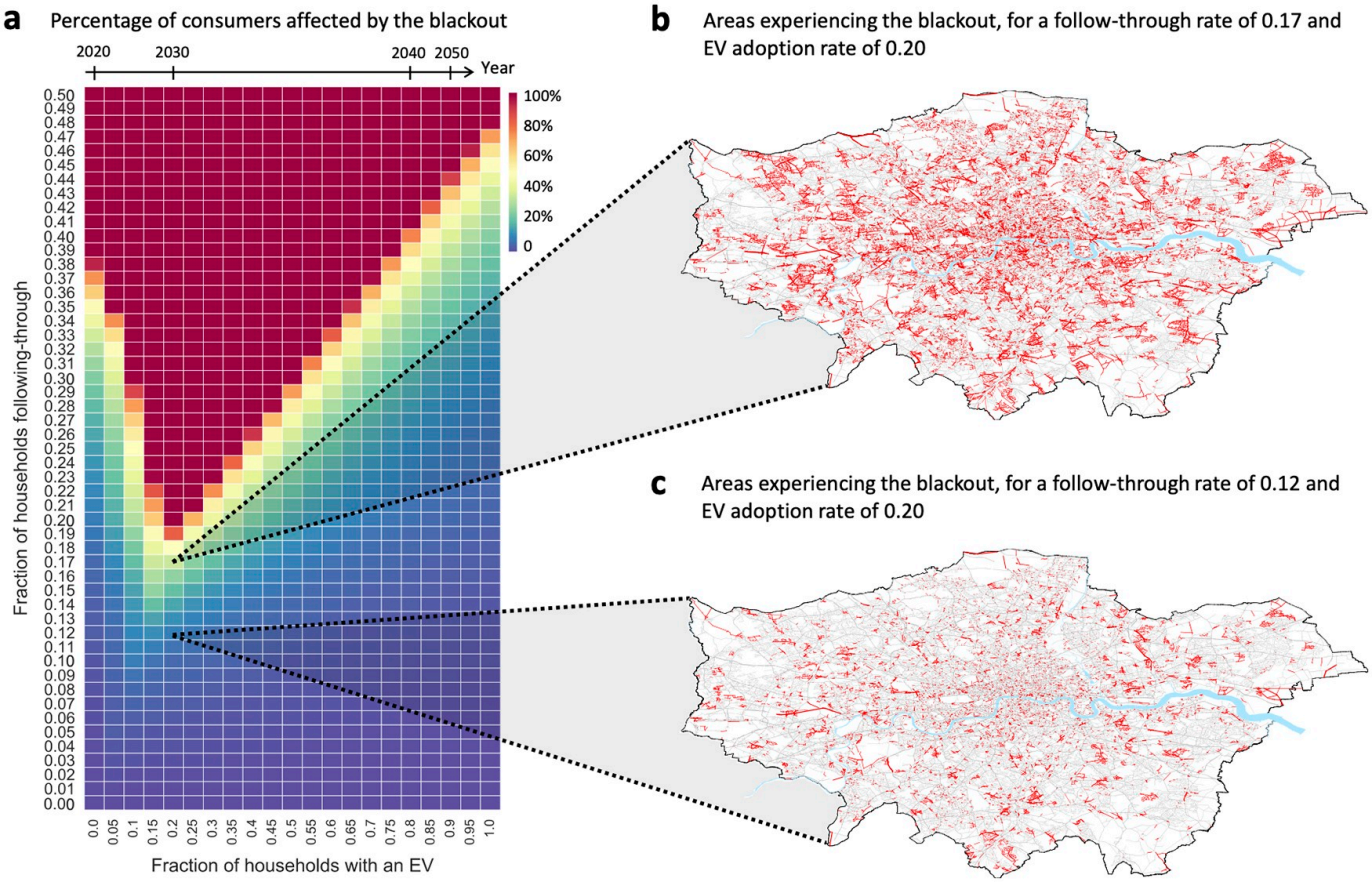
Impact of an attack on the power distribution network of Greater London. a: The percentage of consumers suffering from a blackout as a result of the attack given different follow-through rates and EV adoption rates. The figure also highlights the columns corresponding to projected EV adoption rates for the UK in the years 2020, 2030, 2040, and 2050. b: Visualization of the status of every power distribution line in the system for a follow-through and EV adoption rates of 0.17 and 0.20, respectively. Grey indicates active lines, whereas red indicates lines that have tripped as a result of overloading. c: The same as (b), but for follow-through and EV adoption rates of 0.12 and 0.20, respectively.

We consider a scenario where the grid is heavily loaded and any distribution line can sustain at a maximum a 10% increase in the peak demand through it (see Methods). Fig 2a presents the percentage of consumers who experience a blackout given varying follow-through and EV adoption rates. As can be seen, increasing the EV adoption up to 20% increases the system vulnerability to the attack, whereas beyond 20% the system resilience increases, i.e., it requires a greater follow-through rate to achieve the same attack magnitude. This trend is caused by two opposing forces: (i) increased vulnerability due to the consumers controlling more deferrable energy, and (ii) increased resilience due to the grid’s upgraded capacity to cope with the increased number of EVs. When the EV adoption is smaller than or equal to 20%, the former force outweighs the latter, and hence we see an increase in the system vulnerability. The opposite is true when the EV adoption exceeds 20%, leading to the observed increase in resilience. Next, to get a sense of the distribution of the blackout across the city, we depict the state of the system corresponding to two different cells in the heat map; see Fig 2b and 2c. As can be observed, the impact is dispersed throughout the city rather than being concentrated in very few massive pockets.

We then study how the grid’s vulnerability depends on the peak overloading capacity of the distribution lines. Say the overloading capacity is increased from 10% to 15%. Simulating the system for follow-through and EV adoption rates of 0.17 and 0.20 respectively, we find that the attack results in only 5.9% of consumers being offline. This is in contrast to 35.4% of consumers that were affected by the blackout when the line capacity was 10% (see Fig 2b). Further increasing the overloading capacity to 20% reduces the size of the blackout to 1.4% of the consumers. The heat maps corresponding to these scenarios are presented in S2 Note in S1 File. To obtain more insight, we analyze the grid in terms of the line capacity upgrades that are necessary to support increasing EV adoption. The results shown thus far are for the case where, for any given EV adoption rate, the grid is assumed to be upgraded to support exactly that rate. However, if the grid is upgraded to support more than this rate, the impact of the attack will be substantially alleviated, and vice versa. Taking the year 2025 as an example, if by then the grid was not upgraded since 2020, then a mere 5% follow-through rate can bring the grid down completely. On the other hand, if the grid in 2025 was upgraded to support the projected EV adoption until 2030, then even a 100% follow-through rate would cause a blackout for less than 20% of the residents. These results highlight the need for future grid upgrades to not only be dictated by the technical aspects governed by physical laws, but also consider the behavioral aspects of the consumers who may act unpredictably and irrationally, especially when subjected to disinformation. However, since grid upgrades come at a high cost to the power utility [35], perhaps a more realistic solution would be to focus on increasing the awareness of the consumers and immunizing them against disinformation.

## Estimating disinformation follow-through

**Fig 3:**
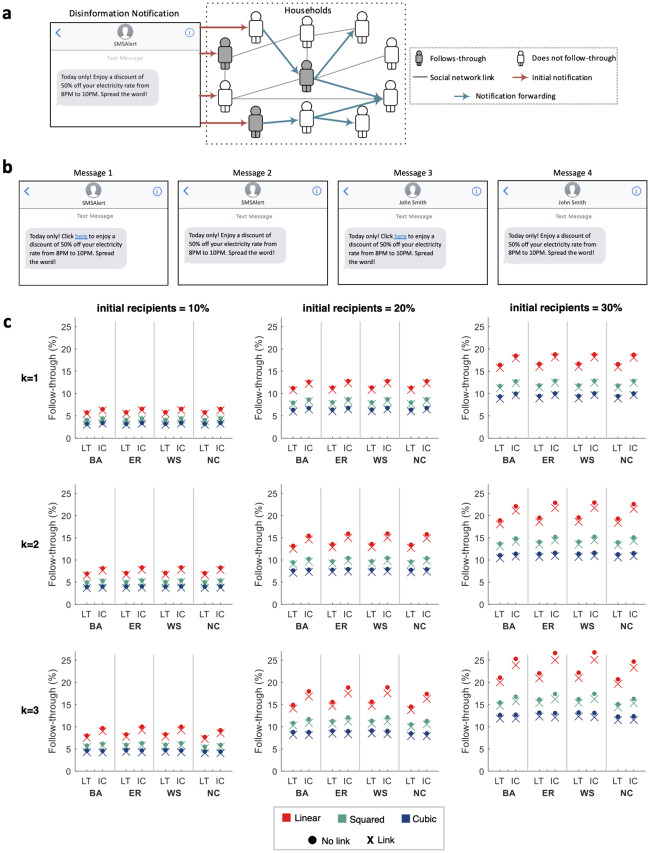
Attack diffusion. a: An illustration of how disinformation can propagate through a social network. b: The disinformation notifications shown to participants in different conditions, which vary depending on whether or not the notification contains an external link, and whether the sender is a stranger (assumed to be the attacker who uses spoofing services to mask the sender as *SMSAlert*) or a friend (named *John Smith* in the survey). c: Given different percentages of initial recipients (10%, 20% and 30%), and different values of *k* (representing the number of friends to whom each recipient considers forwarding the notification), the subfigures depict the follow-through rates after a single step of propagation in social networks consisting of 1 million individuals. The networks were generated using four network models: Barabási-Albert (BA), Erdős-Rényi (ER), Watts-Strogatz (WS), and Newman Configuration (NC). The propagation is simulated using two influence propagation models: independent cascade (IC) and linear threshold (LT). The participants’ propensities to follow-through or forward the notification (which were reported on a Likert scale from 0 to 10) were mapped to actually probabilities (from 0 to 1) using three functions: linear, squared, and cubic. Results are shown for two cases, one where the notification contained an external link (marked as an ‘X’ in the subfigures), and one where it did not (marked as an ‘O’).

Having assessed how the power grid is affected by the consumers who follow-through on the fake notification, we now estimate what follow-through rates could be achieved by an attacker in reality. Here, the social aspect could play an important role, since people may unknowingly amplify the attack by forwarding the disinformation notification to their friends; see Fig 3a for an illustration. (Note that the term “friend” is borrowed from the context of social media to refer to any “acquaintance”.) In this context, Goel et al. [36] analyzed a billion diffusion events, and found that (dis)information is unlikely to become *viral* through social media, since the vast majority of the studied events terminated either right after the initial broadcast itself, or after a single step of propagation through social media. As such, assuming that the attack considered here has similar limitations, our analysis considers only a single step of propagation, whereby the initial recipients of the notification consider forwarding it to some of their friends.

To model the spread of disinformation, we use two standard models of influence propagation, namely, independent cascade [37], and linear threshold [38]. In both models, a person may receive the same notification from more than one friend, but the main difference between the two models lies in the way in which that person is influenced by such repeated exposure to the notification. In the independent cascade model, every exposure has an independent probability to persuade the recipient to modify their behavior—these probabilities constitute the main parameters of the model. In contrast, each person in the linear threshold model has a “threshold” specifying the number of exposures required for them to modify their behavior—these thresholds constitute the main parameters of the model; see Methods for more details. In our simulations, the values of these parameters cannot readily be taken from existing studies since the diffusion models had to be modified to accommodate the specifics of our scenario; see Methods. One possible way to determine the parameters of our models is to use random values, but the resulting model may be unrealistic. Arguably, a more realistic model would be one whose parameters are determined based on survey responses.

Driven by this observation, we surveyed 5,124 participants who were recruited through Amazon Mechanical Turk. Specifically, the participants were shown a message notifying them of a discount of 50% in their electricity rate from 8PM to 10PM. They were then asked to specify the likelihood of them changing their electricity-use patterns to take advantage of this discount, and the likelihood of them forwarding this message to their friends. We tested two factors that may influence the behavior of the participants: (i) the notification sender, and (ii) the notification content. As for the first factor, while such notifications are typically received from the power utility, we analyzed the cases when they are instead received from either a stranger or a friend. We considered these two possibilities since some people may receive the spoofed message directly from the attacker (who is a stranger to them), while others may receive it indirectly through friends who forward it to them. As for the second factor—the notification content—we analyzed two variants: one where the discount can only be availed by clicking on an external link, and another where the discount is unconditional. This manipulation allows us to understand the differences, if any, between the context of phishing and spam attacks—which require the recipients to click on an external link embedded in the message—and the context of our disinformation attack—where no such link is necessary. Accordingly, the participants were randomly assigned to one of four conditions: (i) receive a notification with a link from a stranger; (ii) receive a notification without a link from a stranger; (iii) receive a notification with a link from a friend; (iv) receive a notification without a link from a friend. The corresponding messages that were displayed to participants are depicted in Fig 3b. They were then asked questions to determine how they would react to these messages. Here, participants were further split into two groups depending on the influence model being studied, since the parameters of each model require the questions to be framed differently. The complete survey along with a summary of the results is provided in S3 Note in S1 File.

The research was approved by the Institutional Review Boards of the New York University Abu Dhabi (#025-2019) and the National University of Singapore (ref. S-19-162), and all research was performed in accordance with the relevant guidelines and regulations. Written informed consent was obtained from all survey participants.

Admittedly, the survey instrument is not ideal, as the respondents’ behavior in a real-life situation may not be exactly what is reported in the survey. Nevertheless, it provides important clues to how participants may behave in reality. For example, consider two arbitrary participants *p*_1_ and *p*_2_ that state their follow-through propensity to be *n*_1_ and *n*_2_, respectively, such that *n*_1_ > *n*_2_. It seems reasonable to assume that, in a real-life situation, *p*_1_ is *more likely* to follow-though than *p*_2_. Otherwise, it would be strange to claim that, on average, those who state a greater propensity in the survey are *less likely* to follow-through in real-life. As such, while the participants’ actual propensity is unknown to us, it seems reasonable to claim that there exists some monotonic function that maps the participants’ stated propensity to their actual (unknown) probability to either follow-through or forward the notification. An intuitive candidate for this is a linear mapping function that translates the participants’ response on a Likert scale from 0 to 10 to the corresponding probability in [0, 1], e.g., if a participant states that their propensity is “5”, then their probability to follow-through is simply 50%. In addition to the linear mapping, we also consider squared and cubic alternatives (see Methods), which provide more conservative estimates to reflect the fact that survey participants may over-report their propensity to follow-through or forward the notification, e.g., given a participant who reports their willingness to follow-through to be 5 out of 10, the cubic mapping implies that their probability of following in real-life is only 12.5%.

Next, the probabilities obtained from the survey were used to evaluate the overall follow-through rates that could be achieved by an attacker. To this end, we conducted simulations on 100 randomly generated networks based on four network models—Barabási-Albert [39], Erdős-Rényi [40], Watts-Strogatz [41], and Newman Configuration [42]—with each network consisting of 1 million nodes (see S4 Note in S1 File). For each node in a network, its probability to follow-through and forward the notification is set to match that of a randomly-chosen survey participant. Within our simulations, nodes that receive the notification forward it to *k* of their friends with a probability that corresponds to their respective preferences. For instance, if *k* = 3 and a participant specifies their likelihood of forwarding the message to be 50%, then the corresponding node chooses 3 friends at random, and forwards the notification to each of them with a probability of 50%. Every simulation proceeds in time steps as follows. The nodes that receive the notification in a time step *t* may decide to follow-through (depending on their preferences), and may forward it to their friends (i.e., the other nodes that are connected to them in the network) who would then receive it in time step *t*+ 1. Since we are focusing on a single step of propagation, we analyze the follow-through rates at the end of the first time step, i.e., after the initial recipients have had the chance to forward the notification to their friends.

Given four network models, two influence propagation models, and three values of *k*, Fig 3c depicts the average number of people who follow-through, assuming that the initial notification is sent to 10%, 20% and 30% of the individuals in the network. As can be seen, the final follow-through rates range from 3.2% to 26.8%. The rates could be greater in reality, since our simulations consider *k* ∈ {1, 2, 3}, whereas the value of *k* in reality could be far greater, e.g., if a person considers posting the notification on Facebook or Twitter, then *k* would be equal to the number of people following that person, which could be in the hundreds or even more. Finally, recall that unlike the case of phishing and spam attacks, the disinformation attack considered in our scenario does not require the recipient to click on an external link. To evaluate how this difference affects the impact of the attack, we run similar simulations based on the responses of participants who were shown a message containing an external link. We found that the omission of the link always increases the follow-through rate (see Fig 3c); depending on the model, the increment ranges from 3.4% to 9.8% at the end of one step of propagation.

Now, consider the case when the EV adoption rate in the power grid is 15%. In this case, if 30% of the population were targeted by the attacker initially, then our results in Fig 3c show that the resultant follow-through rate ranges from 9.4% to 26.8%. Our power grid simulations shown earlier in Fig 2a indicate that these follow-through rates would result in a blackout for 5.6% to 100% of the residents, respectively. To put it differently, behavioral manipulation through disinformation can indeed lead to a full blackout in a heavily loaded grid.

## Discussion

While the literature on power grids focuses on the advantages of increasing the active consumer engagement (through demand response programs) and coordinating their consumption patterns [34, 35, 43, 44], we demonstrated that such engagement makes the grid more vulnerable to behavior manipulation attacks. In particular, we showed how an adversary can use disinformation to manipulate the behavior of energy consumers by sending them fake notifications that encourage them to shift their energy usage into the peak demand period. We quantified the impact of such an attack on a city-scale, taking Greater London as an example and considering the additional demand flexibility introduced due to residential EV adoption. We also analyzed how the attack impact varies with the overloading capacity of the distribution lines, showing that heavily loaded grids are particularly vulnerable to such attacks. Our surveys showed that people are willing to not only follow-through on such notifications, but also forward them to their friends, thereby amplifying the attack. This is partly attributed to the fact that such notifications, unlike those used in spam and phishing attacks, do not include any external links in the message. Moreover, consumers who participate in demand response programs and regularly respond to utility requests by changing their demand patterns are likely to believe tailored disinformation notifications, thereby increasing the overall follow-through rate.

Policymakers need to plan effective mitigation strategies to address the vulnerability highlighted in this study. For instance, after the fake notifications are sent out and before a blackout happens, law enforcement and other governmental agencies have a window of opportunity to act, e.g., by broadcasting notices on local TV stations to warn the general public of the attack. In our scenario, to allow for the fake notification to propagate in the social network, the attacker was assumed to send the notification a few hours before the peak demand period, which is the time available for the authorities to act. However, we have shown that just a single step of propagation can result in a high enough follow-through rate to fully blackout the grid. Moreover, if the fake notifications are sent out to a sufficiently large number of people to begin with, then the attacker need not rely on propagation at all. This, in turn, allows the attacker to send the notifications only a few minutes before the peak demand period, thereby reducing the authorities’ window of opportunity even further. As such, it is critical that any such attacks are detected as soon as possible, and any proposed mitigation strategy can be implemented at a short notice.

Our analysis has four main limitations which will be discussed next. First, the responses obtained in our survey may not be fully representative of consumer reactions in reality. One alternative to a survey-based approach would be to run a field experiment where we send fake notifications to actual energy users and assess their responses. Such an experiment would require us to register the reactions of the recipients, e.g., by getting them to click on a link embedded in the notification itself, which would lead them to a website where we can register their actions. However, as we noted earlier, the notification considered in our disinformation scenario does not contain an external link; the absence of such a link renders the experiment futile. Moreover, even if we were able to somehow register whether the recipients follow-through, e.g., by monitoring their energy usage, we will not be able to know whether they have forwarded the notification to their friends. Instead, we opted for a survey-based approach whereby we can register the participants’ propensity to follow-through and forward to their friends, while accounting for the possibility that the participants may have over-reported their propensity in the survey. This was achieved by considering mapping functions that yield conservative probability estimates, e.g., selecting “5” on a Likert scale from 0 to 10 would yield a probability of just 12.5% given the cubic mapping function. Further, we note here that while the experimenter demand effect is a frequent criticism in survey-based approaches, recent studies (e.g., see [45]) suggest that this effect is small, and hence, we do not consider it in our analysis.

Second, our simulations focus on residences while disregarding commercial, industrial, and critical buildings. However, the latter are likely to have their own blackout protection schemes such as the installation of backup generators. This is unlike residences which typically lack such protection, making them especially vulnerable to the attack considered in our study.

Third, we disregard the possibility that power utilities react to the sudden increase in the demand either by increasing the available generation or through load shedding [46]. As for the former reaction, it would be ineffective since the primary cause of the blackout in our simulation is the violation of the line capacity limits rather than a generation deficit. The latter reaction may also be ineffective in protecting the residential loads considered in our study since power utilities typically prioritize commercial, industrial and critical loads during such contingencies.

Finally, our disinformation scenario is not applicable to households equipped with automated energy management systems. Such systems optimize the energy consumption by automatically scheduling appliances based on price signals sent by the power utility. As such, they reduce the need to incentivize residents to change their consumption patterns. Nevertheless, such systems can be inadequate in reducing the system peak demand, and are still uncommon to date (e.g., see [47–49]). In contrast, the schemes that we consider here, which focus on incentivizing people to change their behavior, have proven to be effective and are more widespread among residential consumers [50, 51].

In conclusion, we demonstrated that an adversary can cause blackouts on a city scale, not by tampering with the hardware or hacking into the control systems of the power grid, but rather by focusing entirely on behavior manipulation. On a broader note, our study is the first to demonstrate that in an era when disinformation can be weaponized, system vulnerabilities in critical infrastructure arise not only from the hardware and software, but also from the behavior of the consumers.

## Materials and methods

### Disinformation content

The lines in the power grid have limited capacity that cannot easily be expanded due to financial constraints. Consequently, legacy networks are often highly loaded and unable to support extremely unlikely scenarios, e.g., when the peak demand increases abruptly in a totally unexpected manner as in the scenario considered in our paper. This limitation is further exacerbated by the inclusion of EVs, which have the highest real power consumption compared to household appliances. Given this limitation, an adversary may engineer a scenario aimed at breaching the capacity limits of the power lines. One way to achieve this goal is to persuade as many people as possible to shift their energy consumption into the peak demand period, when the grid is already at its most vulnerable state. In our setting, this is achieved by spreading a fake discount message informing people of a discount effective during the peak period.

The effectiveness of the disinformation in persuading recipients to follow-through depends on its content, which needs to be tailored to fit the schema of legitimate communications that the consumer receives from the power utility. Designing effective disinformation notifications may not be hard for a sophisticated adversary who monitors existing utility-consumer communication channels, and given the fact that utilities routinely publish their consumer engagement strategies online (e.g., see [50]). Further, even in a deregulated scenario, each city is usually served by at most a few utility companies, which allows the adversary to target entire neighbourhoods with a given design of the fake notification. High levels of disinformation follow-through can be expected if the recipients are consumers who are already enrolled in active consumer engagement or demand response programs, and routinely respond to utility requests by changing their energy usage patterns.

### Power grid specifications

Due to its sensitivity, comprehensive data describing the power distribution network of Greater London is not publicly available. We therefore built our own model based on data obtained from OpenStreetMap [52], under the reasonable assumption that power lines (i.e., overhead lines or cables) are laid alongside roads. In particular, we start by extracting the road network and the location of every building therein. Next, we obtain the locations of the 9 high-voltage transmission-level substations that supply the low-voltage power distribution network of Greater London as reported by the transmission line operator, National Grid [53]. Note that each building is electrically connected to a single substation, which is typically the one closest to it. With this in mind, we divide the road network of Greater London into 9 subnetworks, one per substation, and construct 9 spanning trees connecting the buildings within each subnetwork to the corresponding substation. We construct these spanning trees using a modified version of Kruskal’s algorithm [54] while taking into consideration various technical and economical constraints. The edges in these spanning trees represent the power lines in our simulations. As for the loading of each line in the power grid, we assume that every building represents a household, the energy consumption of which is modelled using statistics obtained from [55, 56]. For more details, see S1 and S5 Notes in S1 File.

### Power grid simulations

Since the energy consumption of a residence depends on its occupancy [55], residences in our simulation were assigned occupancy values based on UK National Statistics [56]. As for the EVs, we restrict our attention to residential rather than commercial EVs since the charging times of the former align more closely to the overall system peak demand period [57] and can be easily deferred by the consumers according to the fake notification. In each simulation, EV owners and notification recipients were selected randomly based on the EV adoption rate and disinformation propagation rate in the simulation, respectively. Each resident was assigned a daily load profile depending on whether they own an EV and whether they follow-through on the notification (see S1 Note in S1 File for how these load profiles were generated). Note that in our simulations, the same residents who own an EV in the baseline scenario (i.e., the scenario where no resident receives the fake notification) also own an EV in the attack scenario. Also note that the EV adoption rate is varied in our simulations. As such, the power grid is initially assumed to be capable of supporting the residential demand with no EV adoption. Then, as we increase the EV adoption rate, the grid is “upgraded” to support this EV adoption, and the lines are now capable of supporting the increased EV demand under normal circumstances, i.e., in the baseline scenario (by “upgrade” we simply mean increasing the line capacity). Power flows within the grid were then calculated accordingly.

Finally, we analyze how the distribution network is affected by the attack. In this analysis, among the many variables that could be considered such as voltage and reactive power flows, we focused on line capacity limits since they are the most dominantly affected by peak demand growth, and are critical to power system stability [33, 35]. For the results shown in Fig 2, we make the assumption that the capacity of each line in the distribution network is limited to 10% over the peak power flow in that line under regular circumstances when no resident receives the notification from the attacker. By this, we mean that overcurrent relays are set to trip the distribution lines if the power flows result in currents exceeding the 10% threshold. More formally, we assume that a distribution line is overloaded if the following condition is satisfied:

$$\begin{array}{c} \frac{P_{peak}{}_{,attack}-P_{peak}{}_{,normal}}{P_{peak}{}_{,normal}}>10\%, \end{array}$$ (1)

where *P*_*peak,normal*_ and *P*_*peak,attack*_ represent the peak demand through the line under the normal and attack scenarios, respectively. S2 Note in S1 File analyses four alternative scenarios: three where the threshold is changed to 5%, 15%, and 20%, and one where non-uniform loading across the grid results in thresholds varying between 5% to 15% for the different lines. Once a line over-loads it goes offline, leading all lines below it in the power distribution tree to go offline as well. By averaging over 100 such simulations, we obtained the fraction of residences suffering from a blackout that were depicted in Fig 2a. The illustrations in Fig 2b and 2c represent a single simulation each.

### Models of influence

We use two fundamental models of influence propagation in social networks, namely, independent cascade [37] and linear threshold [38]. Both models start with an “active” subset of nodes, called the *seed set*. Then, the influence of these active nodes propagates through the network in time steps. Formally, let *V* denote the set of nodes in the network, and *N*_*v*_ denote the neighbors of node *v* ∈ *V*. Moreover, let *A*(*t*)âŠ†*V* be the set of active nodes at time step *t*, implying that *A*(1) is the seed set. The mechanism of influence propagation depends on the model being used. In the independent cascade model, every pair of nodes has an activation probability, *p*: *V* × *V* → [0, 1]. Then, in each time step *t* > 1, every node *v* that became active at time step *t* − 1 activates every inactive neighbor *w* ∈ *N*_*v*_\*A*(*t* − 1) with probability *p*(*v*, *w*). The propagation terminates when *A*(*t*) = *A*(*t* − 1). On the other hand, in the linear threshold model, every node *v* ∈ *V* is assigned a threshold, *k*_*v*_, such that 0 ≤ *k*_*v*_ ≤ |*N*_*v*_|. Then, in each time step *t* > 1, every node *v* ∈ *V*\*A*(*t* − 1) becomes active if the following holds: |*A*(*t* − 1) ∩ *N*_*v*_| ≥ *k*_*v*_. Again, the propagation terminates when *A*(*t*) = *A*(*t* − 1).

In our analysis, we disregard the potential effects of negative opinions propagating within the network. This is because the energy consumers who follow-through on the fake notification may realize that they were manipulated only when they do not receive the promised price discount, which happens *after* the propagation process ends and when the power utility generates energy receipts in the monthly billing cycle.

We introduced three modifications to the influence propagation models in order to reflect the attack scenario being considered. First, we decoupled the state of being activated from the state of influencing others. Specifically, being activated in our setting means deciding to follow-through on the notification being received. In contrast, influencing neighbors means forwarding the notification to one’s friends. As such, one may be activated without necessarily influencing others, and vice versa. The second modification involved distinguishing between those who receive the notification from a stranger (who is the attacker in our case) and those who receive it from their friends (who forwarded the notification to them). This distinction matters, since the way in which recipients react to the notification is affected by the identity of the sender, as evident from the outcome of our surveys (see S3 Note in S1 File). The third modification involved allowing the individuals to influence only a subset of their friends. This makes the model more realistic, since people are usually not restricted to forwarding a message to either *all* or *none* of their friends. It should be noted that for an individual to be included in the seed set, it is not sufficient for them to simply receive the notification from the stranger; they have to also decide to forward it to their friends. This is especially important in the linear threshold model, since our definition of the seed set means that the model cannot be parameterized solely based on thresholds; it also needs parameters specifying the likelihood of the nodes to forward a notification received from a stranger.

The influence propagation models used in our simulations were parameterized based on the survey outcomes. In particular, for the independent cascade model, each participant specified (i) their likelihood to follow-through, and (ii) their likelihood to forward the notification, when the participant received it either from a stranger or a friend (note that an individual may first receive the notification from the stranger, and then again from a friend at a later time step). On the other hand, for the linear threshold model, participants specified (i) their likelihood to follow-through and forward the notification when received from a stranger, and (ii) the number of friends that they need to receive the notification from in order for them to follow-through and forward it to others (see S3 Note in S1 File). Participants specified their likelihoods on a Likert scale from 0 to 10. Each response, *x* ∈ [0, 10], was then converted to the probability $\left(\frac{x}{10}\right)$, ($(\frac{x}{10}{)}^{2}$, or $(\frac{x}{10}{)}^{3}$ depending on whether the mapping was linear, squared, or cubic, respectively. Finally, in our simulations, the number of friends that each individual considered forwarding the notification to was determined based on a parameter *k* ∈ {1, 2, 3}.

## Supporting information

S1 File (PDF)
