# Supplementary material for: How weaponizing disinformation can bring down a city’s power grid
Source: PLoS One. 2020 Aug 12;15(8):e0236517. doi: 10.1371/journal.pone.0236517 (PMC7423072; doi:10.1371/journal.pone.0236517)
Supplement: S1 File — (PDF) [file pone.0236517.s001.pdf]

Supplementary Materials for

How weaponizing disinformation can bring down a city's power grid

Gururaghav Raman, Bedoor AlShebli[†], Marcin Waniek[†],
Talal Rahwan[*], and Jimmy Chih-Hsien Peng[*]

[*] Corresponding authors. E-mail: talal.rahwan@nyu.edu, jpeng@nus.edu.sg
[†] Equally contributing authors

This document is structured as follows:

- **Section S1** (*page 2*) describes how the power distribution network of Greater London is obtained;
- **Section S2** (*page 4*) describes how the attack impact on the power grid varies with the overloading capacity of the power lines;
- **Section S3** (*page 6*) presents the survey questionnaire and results;
- **Section S4** (*page 15*) illustrates how the follow-through rate varies with the social network model, the influence propagation model, the mapping from the survey participants' stated propensities to the actual probabilities used in the simulations, the number of initial recipients of the notification, and the maximum number of friends to whom the notification can be forwarded;
- **Section S5** (*page 28*) describes how the residential load profiles are generated.

S1 Supplementary note 1: Power distribution network

This section describes the steps that we took to extract the power distribution network of Greater London based on data obtained from OpenStreetMap (OSM) as well as data about the location of the main transmission-level substations therein. The steps are as follows:

1. Construct the road network of Greater London by extracting from OSM all ways such that the value of the *highway* key belongs to the following list: *motorway*, *trunk*, *primary*, *secondary*, *tertiary*, *unclassified*, *residential*, and *service* (the types of the roads are ordered from the most important to the least important according to OpenStreetMap Wiki [1]). We assign the weight of each edge based on the order of this list, with the highest weight assigned to the edges tagged as *motorway*, and the lowest weight assigned to the edges tagged as *service*.

2. Merge nearby nodes—defined here as being within 20 meters of each other—into a single node. This is based on the common practice implemented when designing the distribution infrastructure, wherein only a limited number of power junction boxes are installed by the utility along a street or a distribution line, and every building is fed by the box that is closest to it. Let $G = (V, E)$ be the network before merging any nodes, where V is the set of nodes and E is the set of edges, and $G' = (V', E')$ be the network after merging the nodes. Now, suppose we merged a group of nodes $X \subset V$ into a single node, $x \in V'$, and merged another group $Y \subset V$ into a node $y \in V'$. Then, we have $(x, y) \in E'$ if $\exists v \in X, u \in Y : (v, u) \in E$, in which case we have: $\mathit{weight}(x, y) = \max_{(v,u)\in E: v\in X, u\in Y} \mathit{weight}(v, u)$.

3. Divide the city into regions, each of which is fed by a different transmission-level substation. Taking Greater London as an example, National Grid UK [2] presents the locations of the nine 400 kV/230 kV substations feeding the county. Since each substation feeds a separate region, we construct 9 distribution subnetworks, each of which originates from a different substation to supply a separate region with electricity. To this end, we assign each node $v \in G'$ to the substation closest it geographically. We then divide G' into 9 subnetworks, each of which is induced by the nodes assigned to the respective substation. In what follows, we will refer to these subnetworks as *district networks*.

4. Ensure the connectivity of each district network by repeating the following process: (i) select two disconnected components $G_1 = (V_1, E_1)$ and $G_2 = (V_2, E_2)$ from the district network such that the geographical distance between the closest pair of nodes $x \in V_1$ and $y \in V_2$ is minimal; (ii) add the edge (x, y) to E', and set its weight to the maximal value, i.e., as if it was tagged as *motorway* in the original dataset. This process is repeated until the district network becomes connected. Note that this step is necessary since the OSM road network is often disconnected (due to the fact that OSM map data is crowdsourced and some roads may be left unreported), whereas the power distribution network has to be connected.

5. For each district network, keep only the edges belonging to a *maximum spanning tree*, i.e., a spanning tree that maximizes the sum of edge weights, which is computed using Kruskal's algorithm [3]. The algorithm is adjusted to prefer edges with higher weights, to reflect the fact that power lines tend to be placed along the main roads, rather than smaller streets.

6. Generate information about buildings in the area by extracting from OSM all the nodes, ways and relations tagged with the *building* key. For ways and relations, we set the geographical location of the building to be the average location of all nodes belonging to it; for nodes, we simply use the location of the nodes. For each building identified in OSM, we only consider one household. While this may not always be true, the aim of this exercise is to identify any overloads in the power transmission lines, and by taking the baseline power flow as that of a single residence, we obtain a reasonable approximation of the overloads caused by the attack, which suffices for the purpose of our study.

7. Assign each building to the edge in the district network that is geographically closest to that building. The information about buildings can be documented by storing for each edge in the district network the number of buildings to which it is the closest edge.

8. To make the computation and data storage more efficient, contract the edges in each district network, i.e., iteratively remove every node that has exactly two neighbors. For example, if node y is connected only to nodes x and z in the spanning tree, then replace the edges (x, y) and (y, z) with the edge (x, z). The weight of the new edge is set to be: $\mathit{weight}(x, z) = \max(\mathit{weight}(x, y), \mathit{weight}(y, z))$, while the number of the buildings assigned to (x, z) is set to be the sum of the number of buildings assigned to (x, y) and (y, z).

9. Finally, the root of each district network is taken to be the node that is geographically closest to the substation assigned to that subnetwork.

The power network topology for Greater London obtained using the above steps is illustrated in Fig. S1. This topology, which is used to generate the results shown in the article, is publicly available at [4].

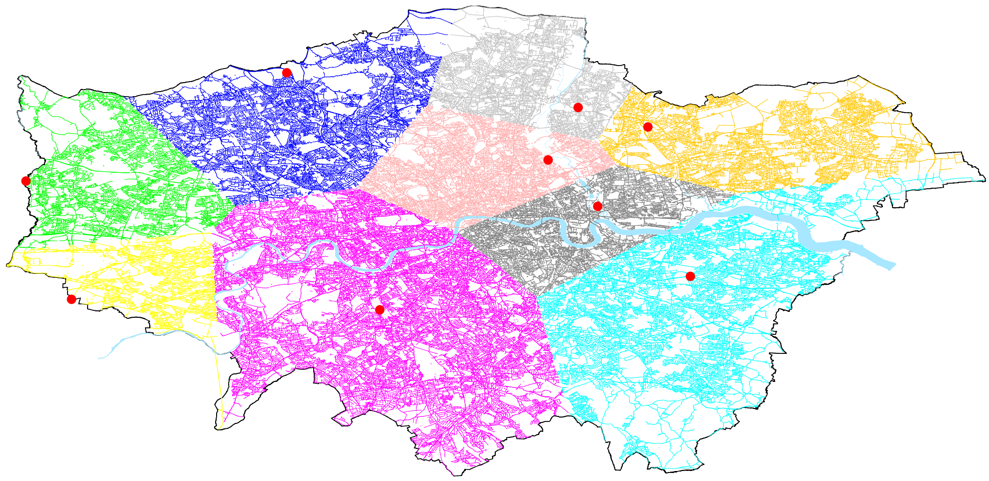

Figure S1: The power distribution network of Greater London, being fed by 9 transmission-level substations. Each color on the map corresponds to a different district network. The red dots indicate the nodes corresponding to the substations.

S2 Supplementary note 2: Variation of the overloading capacity of the power grid

This section describes how the impact of the attack on the power grid varies with the overloading capacity of the power lines. This capacity is defined by a threshold specifying the maximum permissible increase in the peak power flowing through each line in the grid. Lines exceeding this threshold will be tripped offline by protection relays, resulting in all downstream nodes experiencing a blackout.

In Fig. 2a in the main article, this threshold was considered to be 10%. Figs. S2(a)—(c) illustrate the attack impact on the power grid when the threshold is changed to 5%, 15%, and 20%, respectively. To model a scenario where different sections of the grid are loaded differently, Fig. S2(d) illustrates the case when the threshold values for the lines in the network vary, uniformly at random, from 5% to 15%.

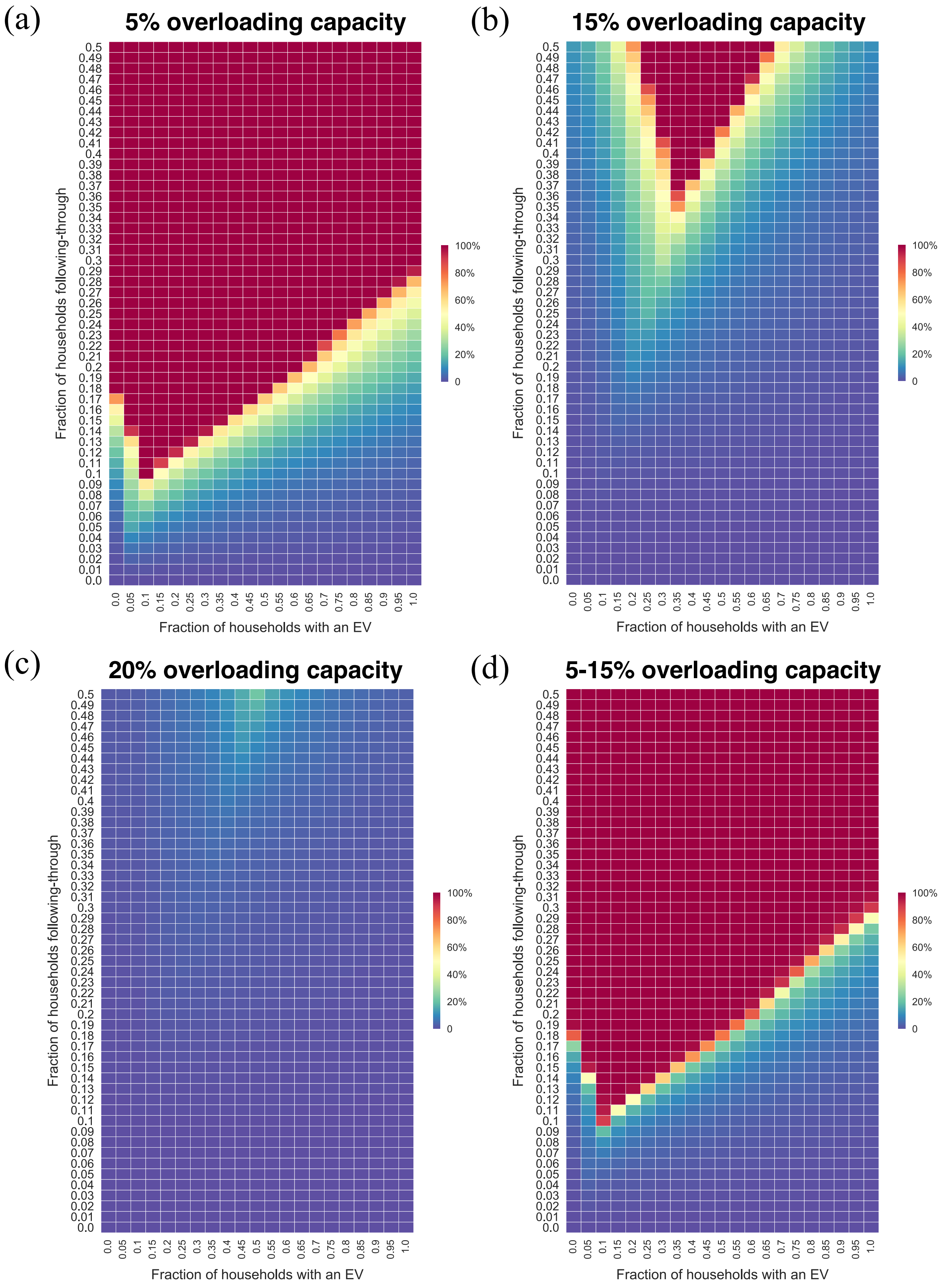

Figure S2: The same as in Fig. 2a in the main article, but when the threshold is changed from 10% to (a) 5%, (b) 15%, (c) 20%, and (d) threshold values for the lines vary uniformly at random from 5% to 15%.

S3 Supplementary note 3: Survey questionnaire and results

To understand how people react to messages containing fake notifications, we recruited 5,124 participants through Amazon Mechanical Turk, who were then directed to a survey on the Qualtrics platform. The participants were residents of one of the following 5 cities in the US: Los Angeles (CA), New York City (NY), San Diego (CA), San Francisco (CA), and San Jose (CA). These cities were chosen since they experience the highest cumulative electric vehicle (EV) sales in the US [5]. The survey consists of five steps, some of which involve multiple options, resulting in various possible "paths" that the participant can follow; see Fig. S3 for a flowchart illustrating those paths. Note that each participant is assigned exactly one path, which is chosen uniformly at random. An overview of the five steps is provided below, whereas a detailed explanation is provided in the Sections S3.2 through S3.5. Finally, the results of the survey are presented in Section S3.6.

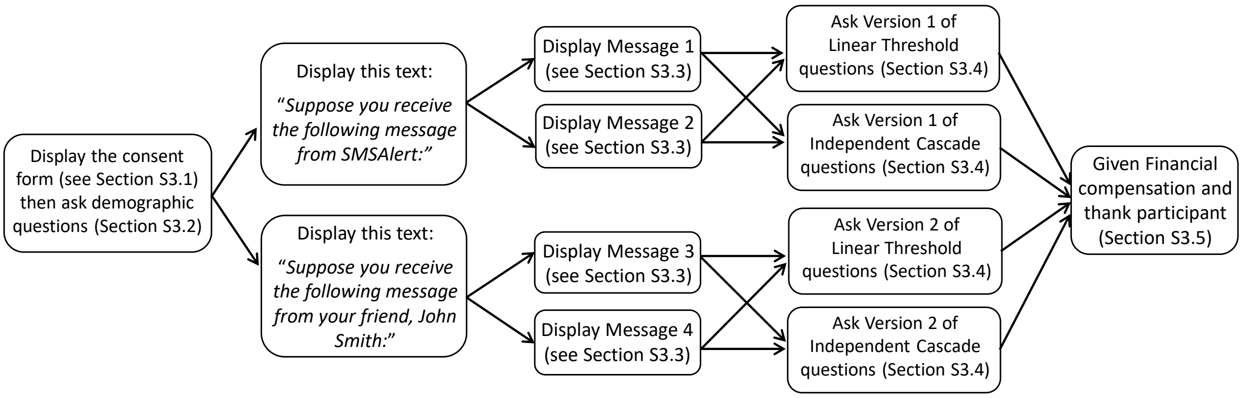

Figure S3: A flowchart illustrating the possible paths in our survey; each participant is assigned exactly one path, chosen uniformly at random.

Step 1: A consent form is displayed (see Section S3.1), after which the demographic questions are asked (see Section S3.2);

Step 2: One of the following sentences is displayed:

- "*Suppose you receive the following message from SMSAlert*";
- "*Suppose you receive the following message from your friend, John Smith*".

Step 3: One of four possible messages is displayed, each containing a fake notification (see Section S3.3). An overview of those messages is provided below:

- **Message 1:** The sender is specified as "*SMSAlert*", and the notification asks the recipient to click on a link;
- **Message 2:** The sender is specified as "*SMSAlert*", and the notification does not contain a link to be clicked;
- **Message 3:** The sender is specified as "*John Smith*", and the notification asks the recipient to click on a link;
- **Message 4:** The sender is specified as "*John Smith*", and the notification does not contain a link to be clicked.

Step 4: The participant is asked one of 4 possible sets of influence propagation questions (Section S3.4);

Step 5: The participant is awarded a financial compensation of \$0.5, and thanked for their participation (Section S3.5).

S3.1 Consent form

The consent form consists of the following text:

> *Welcome to this study investigating how humans behave upon receiving text notifications. You are eligible to participate in the study at this time if you are:*

1. *18 years of age or older;*
2. *live in one of the following cities: Los Angeles, New York City, San Diego, San Francisco, or San Jose.*

The questionnaire asks about your background and your reaction upon receiving text notifications. This survey is anonymous, i.e., it does not contain individually identifiable data from you. Your participation is voluntary, and you may close the survey at any point.

The questionnaire is expected to last on average 5 minutes. An amount of $0.5 will be paid upon successful completion of the survey.

This research is conducted by Jimmy Chih-Hsien Peng and Gururaghav Raman at the National University of Singapore, as well as Talal Rahwan, Bedoor AlShebli, and Marcin Waniek at New York University Abu Dhabi, and has been approved by the respective Institutional Review Boards. For questions about the rights of research participants, you may contact the University Committee on Activities Involving Human Subjects, New York University Abu Dhabi, irbnyuad@nyu.edu and the National University of Singapore, irb@nus.edu.sg.

If you have any questions, suggestions or concerns, please feel free to reach out to us at nyuad.textnotification@nyu.edu – an email address that only researchers associated with this project have access to.

Please do not complete the survey more than once. Upon finishing the survey you will receive a completion code. The payment of $0.5 will be made once you've entered that code in the space provided. ***Please do not close the browser with your MTurk account****. If you read this consent form, and would like to participate in this study, press the button below!*

S3.2 Demographic questions

The demographic questions that are asked to each participant are detailed below:

1. *What is the sex listed on your birth certificate?*
   (a) *Male*
   (b) *Female*
2. *What is your ethnicity?*
   (a) *Hispanic*
   (b) *Non-Hispanic*
3. *What is your race?*
   (a) *White*
   (b) *Black or African American*
   (c) *Asian*
   (d) *Native American*
   (e) *Middle Eastern or North African*
   (f) *Mixed*
   (g) *Other*
4. *What is your age?*

   [empty field to be filled by a number]
5. *What is your highest completed level of education?*
   (a) *Less than high school*
   (b) *High school graduate or equivalent (e.g., GED)*
   (c) *Some college*

(d) *2 year degree (i.e. Associate's degree)*
(e) *4 year degree (i.e. Bachelor's degree)*
(f) *Masters or Professional degree (i.e. MBA, MPP, etc)*
(g) *Doctoral Degree*

6. *What best describes your employment situation?*
   (a) *Full-time employed*
   (b) *Part-time employed*
   (c) *Unemployed*
   (d) *Caregiver (e.g., children, elderly) or homemaker*
   (e) *Retired*
   (f) *Full-time student*
   (g) *Other*

7. *What was your yearly personal income in 2018 (include salary, interests, returns on investments, etc)?*
   (a) *Less than $10,000*
   (b) *$10,000-$19,999*
   (c) *$20,000-$29,999*
   (d) *$30,000-$39,999*
   (e) *$40,000-$49,999*
   (f) *$50,000-$59,999*
   (g) *$60,000-$69,999*
   (h) *$70,000-$79,999*
   (i) *$80,000-$99,999*
   (j) *$100,000-$119,999*
   (k) *$120,000-$149,999*
   (l) *$150,000-$199,999*
   (m) *$200,000 - more*

8. *Which city do you live in?*
   (a) *Los Angeles, CA*
   (b) *New York City, NY*
   (c) *San Diego, CA*
   (d) *San Francisco, CA*
   (e) *San Jose, CA*
   (f) *Other:* [text box appears]
   (if "Other" selected, participant is not eligible and is thanked and exits the survey)

S3.3 Messages with fake notifications

One of the following sentences is chosen and displayed to the participant:

- "*Suppose you receive the following message from SMSAlert*";
- "*Suppose you receive the following message from your friend, John Smith*".

Below the chosen sentence, one of four possible messages is displayed; see Fig. S4. As can be seen:

- **Message 1:** The sender is specified as "*SMSAlert*", and the notification asks the recipient to click on a link;
- **Message 2:** The sender is specified as "*SMSAlert*", and the notification does not contain a link to be clicked;

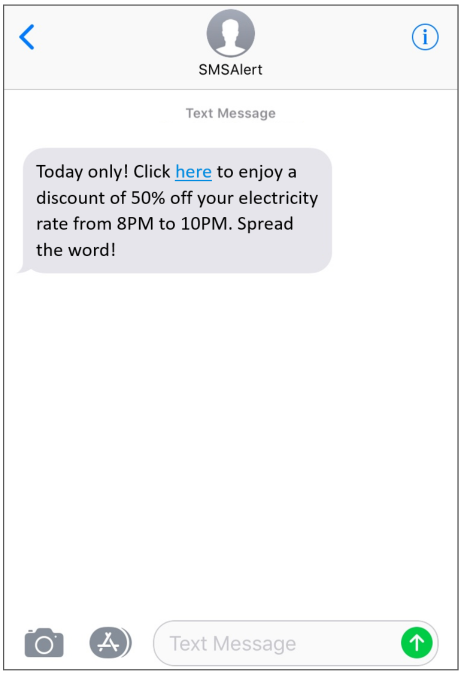

(a) Message 1

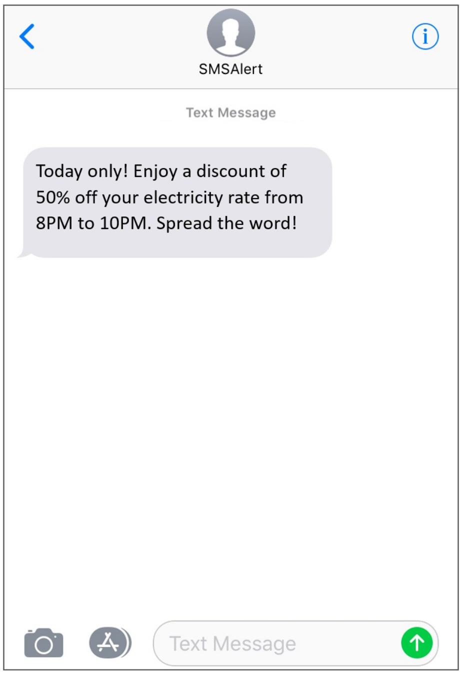

(b) Message 2

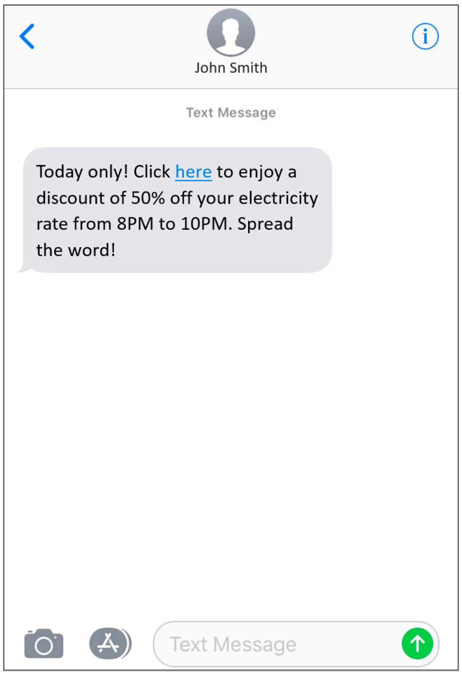

(c) Message 3

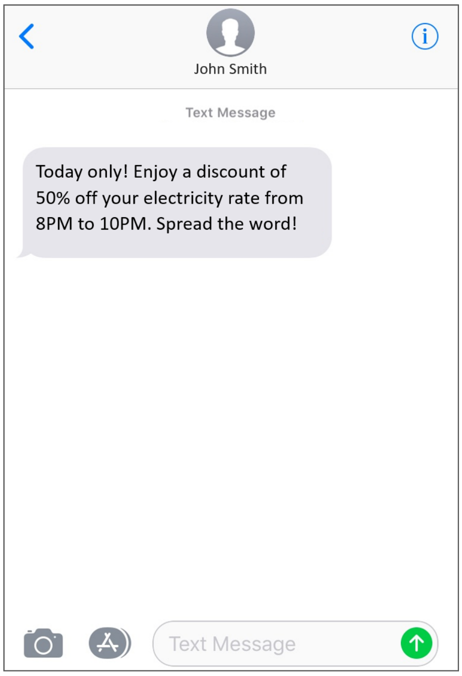

(d) Message 4

Figure S4: The four possible messages used in our survey, each containing a fake notification. Messages 3 and 4 are similar to Messages 1 and 2, respectively, with the only exception being that the sender is specified as “John Smith” instead of “SMSAlert”.

- **Message 3:** The sender is specified as "*John Smith*", and the notification asks the recipient to click on a link;
- **Message 4:** The sender is specified as "*John Smith*", and the notification does not contain a link to be clicked.

As illustrated in Fig. S3, if we display: "*Suppose you receive the following message from SMSAlert*", then either Message 1 or Message 2 is displayed (note that the sender of these messages is specified as "*SMSAlert*"). On the other hand, if we display: "*Suppose you receive the following message from your friend, John Smith*", then either Message 3 or Message 4 is displayed (since the sender of these messages is specified as "*John Smith*").

S3.4 Influence propagation questions

The survey involves four sets of influence propagation questions, determined based on the influence model being studied (either linear threshold or independent cascade) and the sender of the fake notification (either SMSAlert or John Smith):

- **Version 1 of the independent cascade:** asked when studying the independent cascade model given a participant who sees a fake notification sent by "SMSAlert";
- **Version 2 of the independent cascade:** asked when studying the independent cascade model given a participant who sees a fake notification sent by "John Smith";
- **Version 1 of the linear threshold:** asked when studying the linear threshold model given a participant who sees a fake notification sent by "SMSAlert";
- **Version 2 of the linear threshold:** asked when studying the linear threshold model given a participant who sees a fake notification sent by "John Smith".

Note that each participant is asked one set of questions only. Next, we specify the questions that are included in each set.

S3.4.1 Independent cascade questions:

Version 1 (asked when the sender of the fake notification is "SMSAlert"):

1. *What is the likelihood that you change your electricity consumption to take advantage of this discount?*

   [Likert scale with values: $0, 1, \ldots, 10$, where "0" is labeled at "never" and "10" is labeled as "definitely"]

2. *What is the likelihood that you forward this message to your friends?*

   [Likert scale with values: $0, 1, \ldots, 10$, where "0" is labeled at "never" and "10" is labeled as "definitely"]

3. *Suppose that later on you receive the same message, but from your friend, John Smith. What is the likelihood that you change your electricity consumption to take advantage of this discount?*

   [Likert scale with values: $0, 1, \ldots, 10$, where "0" is labeled at "never" and "10" is labeled as "definitely"]

4. *What is the likelihood that you forward the message from your friend, John Smith, to your other friends?*

   [Likert scale with values: $0, 1, \ldots, 10$, where "0" is labeled at "never" and "10" is labeled as "definitely"]

Version 2 (asked when the sender of the fake notification is "John Smith"):

1. *What is the likelihood that you change your electricity consumption to take advantage of this discount?*

   [Likert scale with values: $0, 1, \ldots, 10$, where "0" is labeled at "never" and "10" is labeled as "definitely"]

2. *What is the likelihood that you forward the message from your friend, John Smith, to your other friends?*

   [Likert scale with values: $0, 1, \ldots, 10$, where "0" is labeled at "never" and "10" is labeled as "definitely"]

S3.4.2 Linear threshold questions:

Version 1 (asked when the sender of the fake notification is "SMSAlert"):

1. *What is the likelihood that you change your electricity consumption to take advantage of this discount?*

   [Likert scale with values: $0, 1, \ldots, 10$, where "0" is labeled at "never" and "10" is labeled as "definitely"]

2. *What is the likelihood that you forward this message to your friends?*

   [Likert scale with values: $0, 1, \ldots, 10$, where "0" is labeled at "never" and "10" is labeled as "definitely"]

3. *Suppose that later on you receive the same message, but from your friend, John Smith. Would you change your electricity consumption to take advantage of this discount?*

   If no, would receiving the message from more friends make you change your electricity consumption? If so, specify the minimum number of additional friends that need to send you this message to make you change your electricity consumption.

   (a) *Yes, the message from John Smith is enough.*
   (b) *No, but I am willing to, if the number of additional friends that send me this message is at least:* [Field to enter number].
   (c) *No, I am not willing to, regardless of the number of friends that send me this message.*

4. *Would you forward the message to your other friends?*

   If no, would receiving the message from more friends make you forward the message? If so, specify the minimum number of additional friends that need to send you this message to make you forward the message to your other friends.

   (a) *Yes, the message from John Smith is enough.*
   (b) *No, but I am willing to, if the number of additional friends that send me this message is at least:* [Field to enter number].
   (c) *No, I am not willing to, regardless of the number of friends that send me this message.*

Version 2 (asked when the sender of the fake notification is "John Smith"):

1. *Would you change your electricity consumption to take advantage of this discount?*

   If no, would receiving the message from more friends make you change your electricity consumption? If so, specify the minimum number of additional friends that need to send you this message to make you change your electricity consumption.

   (a) *Yes, the message from John Smith is enough.*
   (b) *No, but I am willing to, if the number of additional friends that send me this message is at least:* [Field to enter number].
   (c) *No, I am not willing to, regardless of the number of friends that send me this message.*

2. *Would you forward the message to your other friends?*

   If no, would receiving the message from more friends make you forward the message? If so, specify the minimum number of additional friends that need to send you this message to make you forward the message to your other friends.

   (a) *Yes, the message from John Smith is enough.*
   (b) *No, but I am willing to, if the number of additional friends that send me this message is at least:* [Field to enter number].
   (c) *No, I am not willing to, regardless of the number of friends that send me this message.*

S3.5 Closing message and compensation

This is the end of the study. Thank you for your participation. Please make note of the following 7-digit code. You will input it through Mechanical Turk to indicate your completion of the study. ***Then click the button on the bottom of this page to submit your answers. You will not receive credit unless you click this button.***

7-Digit Code: [code appears]

S3.6 Survey results

The results from our survey are summarized in Fig. S5 and S6.

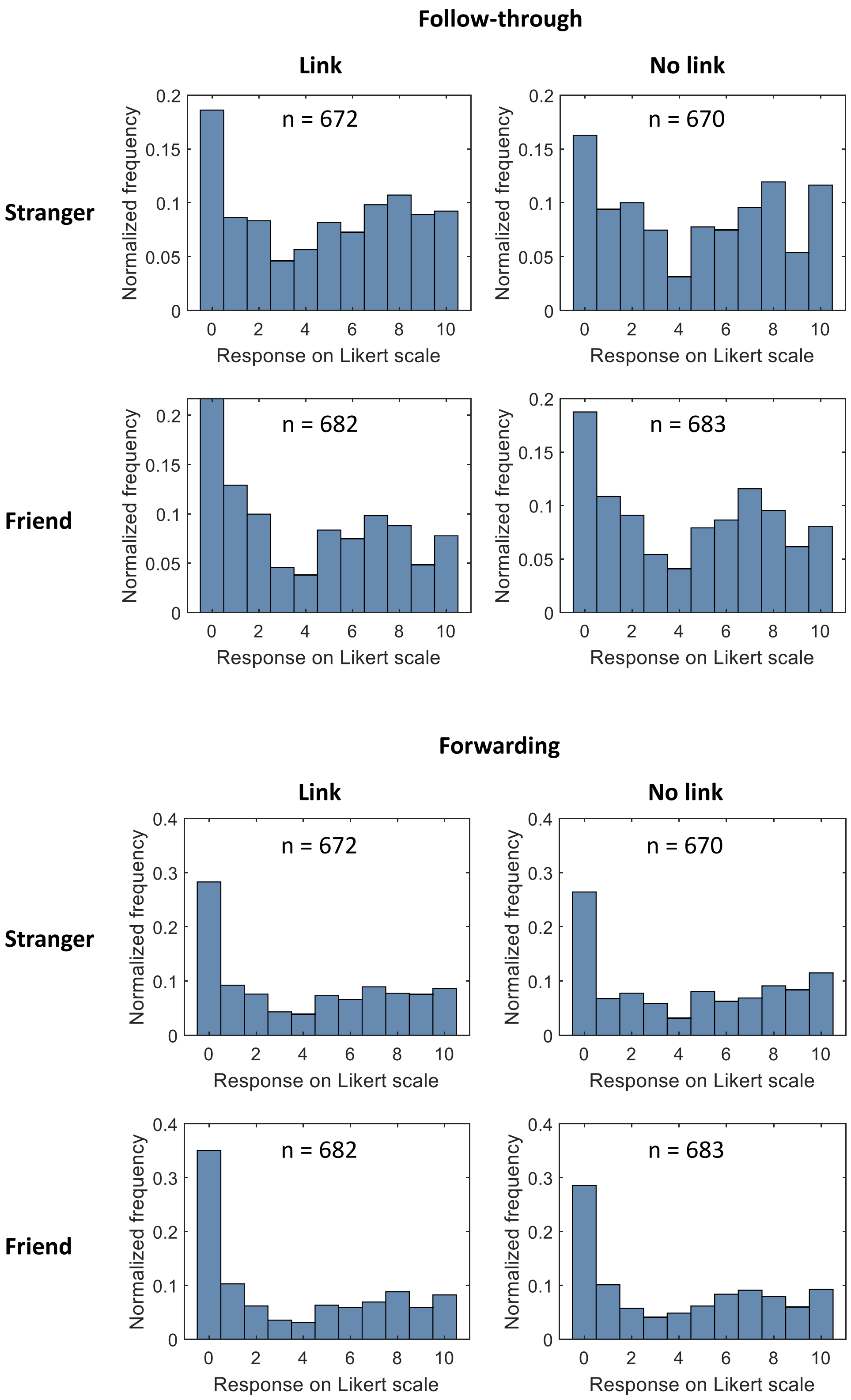

Figure S5: **Survey responses for the independent cascade questions.** A summary of the responses indicating the propensity of following-through and forwarding the notification sent by a stranger vs. a friend, with a link vs. no link. Here, n denotes the number of data points.

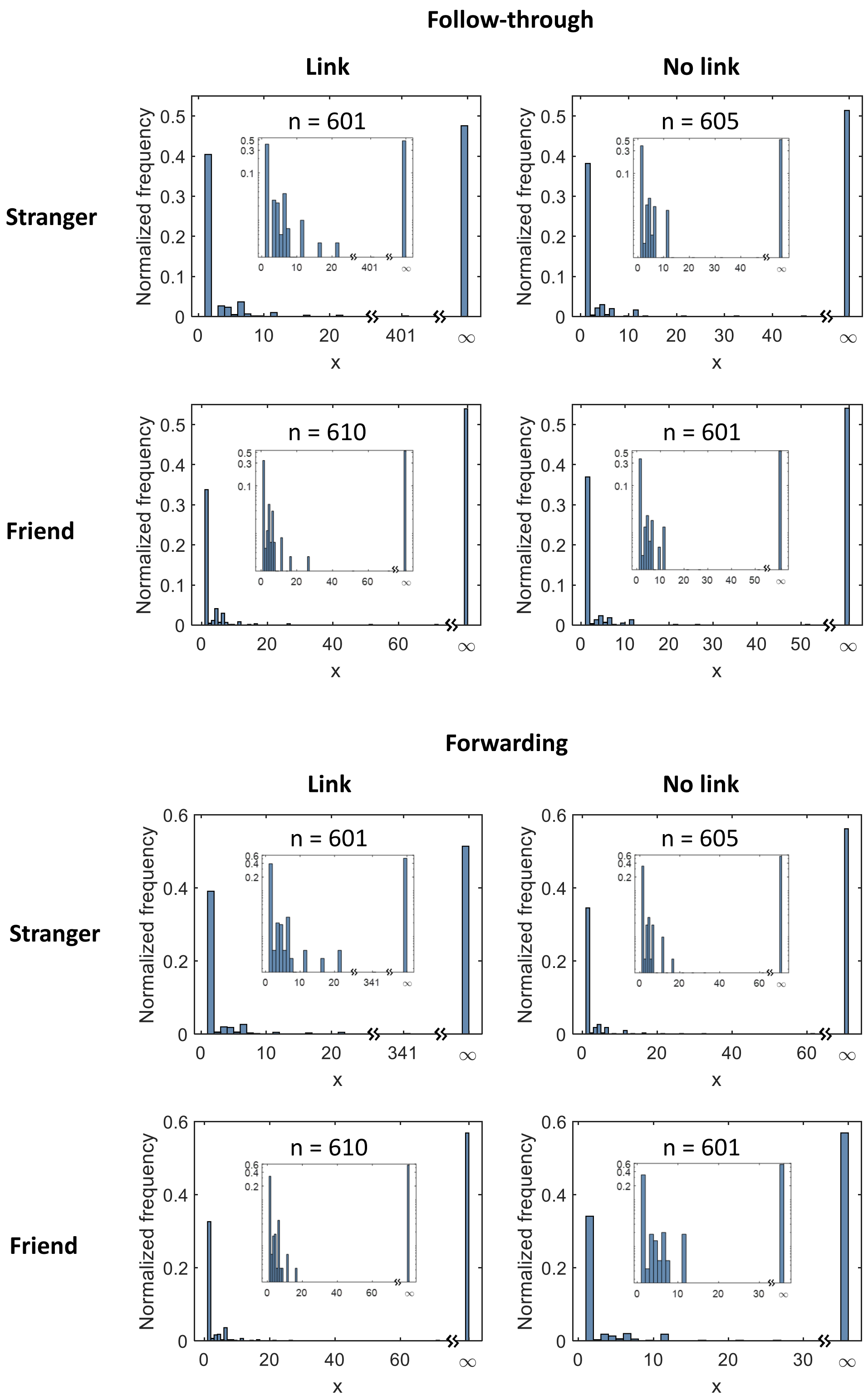

Figure S6: **Survey responses for the linear threshold questions.** A summary of the responses indicating the propensity of following-through and forwarding the notification sent by a stranger vs. a friend, with a link vs. no link. The depicted values are the "thresholds" indicated by the participants, which is the number of friends x required to send the notification to the participant in order for him/her to follow-through and forward it to others. Note that $x = \infty$ represents the case where participants select the option *"No, regardless of number of friends"*. The inset figures show the same data with a logarithmic scale on the Y axis. Here, n denotes the number of data points.

S4 Supplementary note 4: Varying the social network structure and the number of initial recipients

In this section we evaluate the effects of the model used to generate the structure of the social network, the influence propagation model, the mapping from the survey participants' stated propensities to the actual probabilities used in the simulations, and the number of initial recipients of the notification on the follow-through rate. We consider the following network models:

- *Preferential attachment* networks generated using the Barabási-Albert model [6]. In our experiments we add 5 links with each new node and set the size of the initial clique to 5. The results for Barabási-Albert networks are presented in Fig. S7-S9;
- *Random graphs* generated using the Erdős-Rényi model [7]. In our experiments we assume the expected average degree to be 10. The results for Erdős-Rényi networks are presented in Fig. S10-S12;
- *Small-world* networks generated using the Watts-Strogatz model [8]. Unless stated otherwise, in our experiments we assume the expected average degree to be 10 and the probability of rewiring to be $\frac{1}{4}$. The results for Watts-Strogatz networks are presented in Fig. S13-S15;
- *Scale-free* networks generated using the Newman Configuration model [9]. In our experiments we assume the minimal degree to be 2, the maximal degree to be 20, and the configuration model parameter to be $\lambda = 3$. The results for Newman Configuration networks are presented in Fig. S16-S18.

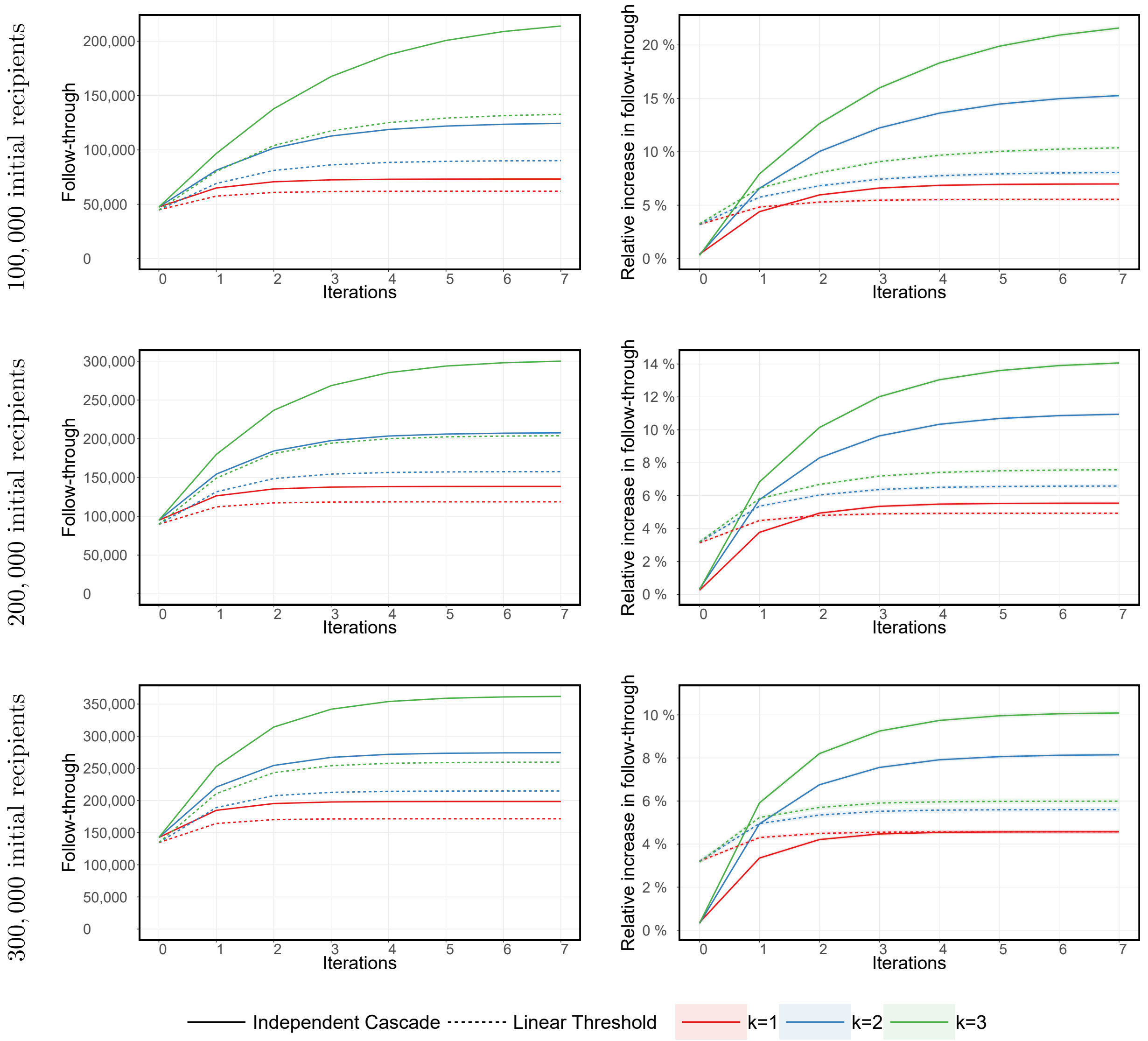

Figure S7: Simulating the spread of the attack in a **Barabási-Albert** social network with 1 million nodes for 100,000, 200,000 and 300,000 initial recipients of the notification, assuming a **linear** mapping from the survey participants' stated propensities (on a Likert scale from 0 to 10) to the actual probabilities (in $[0, 1]$) of them following-through or forwarding the notification. The left column presents the number of individuals following-through when the notification does not contain a link, while the right column presents the relative increase in follow-through as a result of omitting the link. In each plot, the X-axis indicates the time step. The different line types (solid and dashed) represent either the independent cascade or linear threshold influence propagation models, whereas the different line colors (red, blue, and green) represent the number of neighbors k that an individual considers forwarding the message to. Results are presented as an average over 100 simulations, with a new network generated at the beginning of each simulation. Colored areas (very thin) represent the 95% confidence intervals.

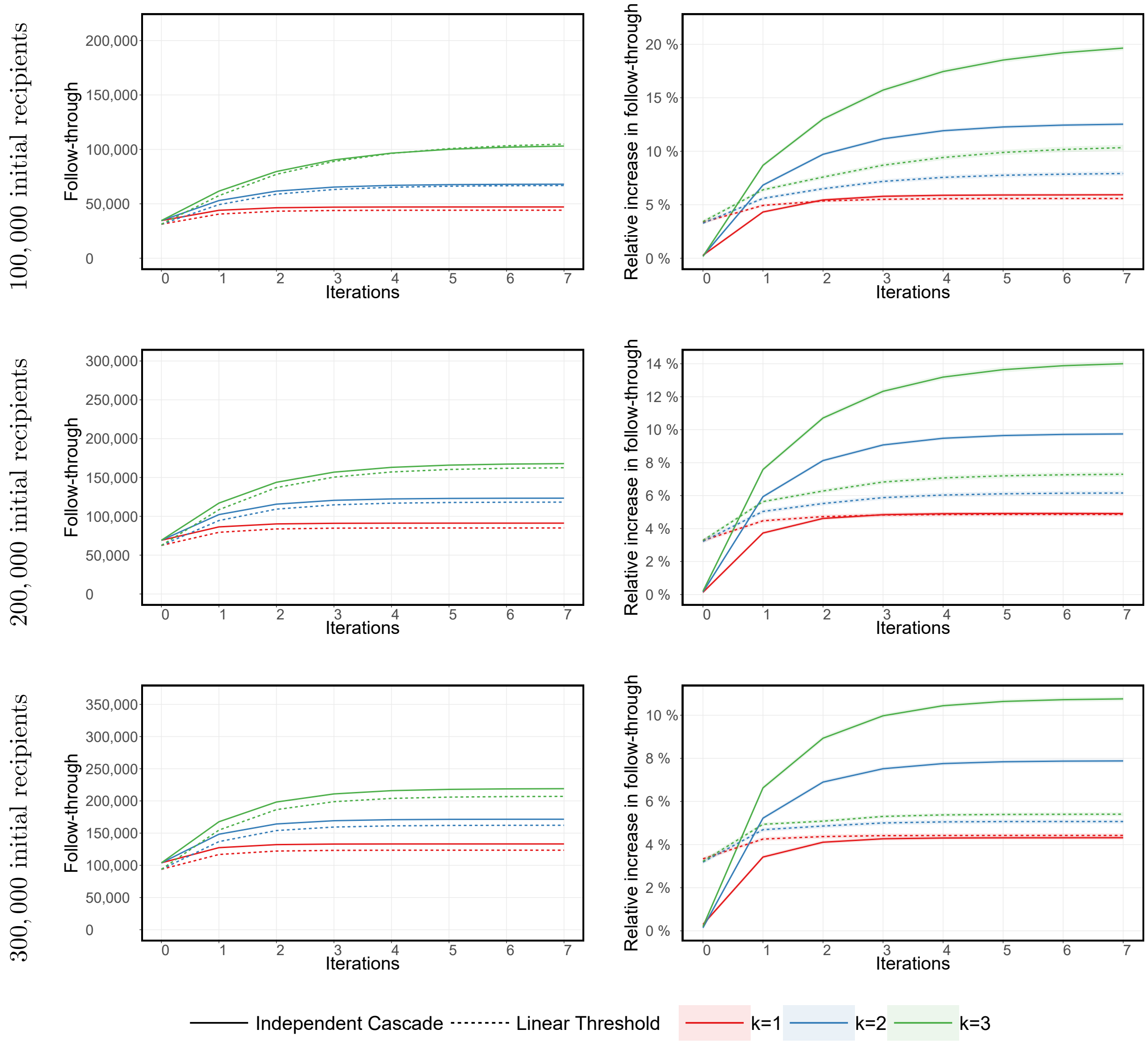

Figure S8: The same as Fig. S7, but for **Barabási-Albert** networks with a **squared** mapping from the participants' stated propensities (on a Likert scale from 0 to 10) to the actual probabilities (in $[0, 1]$) of them following-through or forwarding the notification.

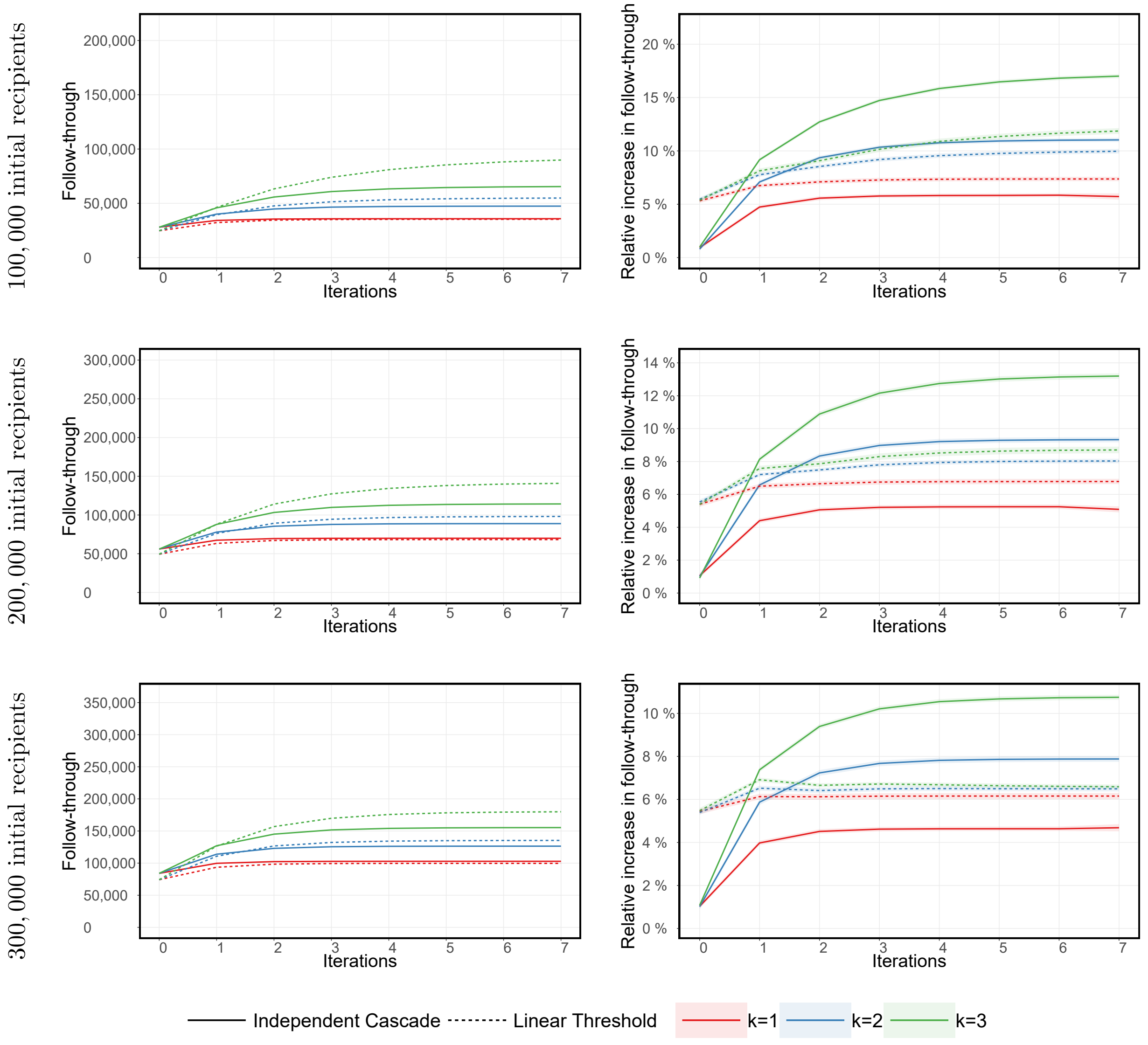

Figure S9: The same as Fig. S7, but for **Barabási-Albert** networks with a **cubic** mapping from the participants' stated propensities (on a Likert scale from 0 to 10) to the actual probabilities (in $[0, 1]$) of them following-through or forwarding the notification.

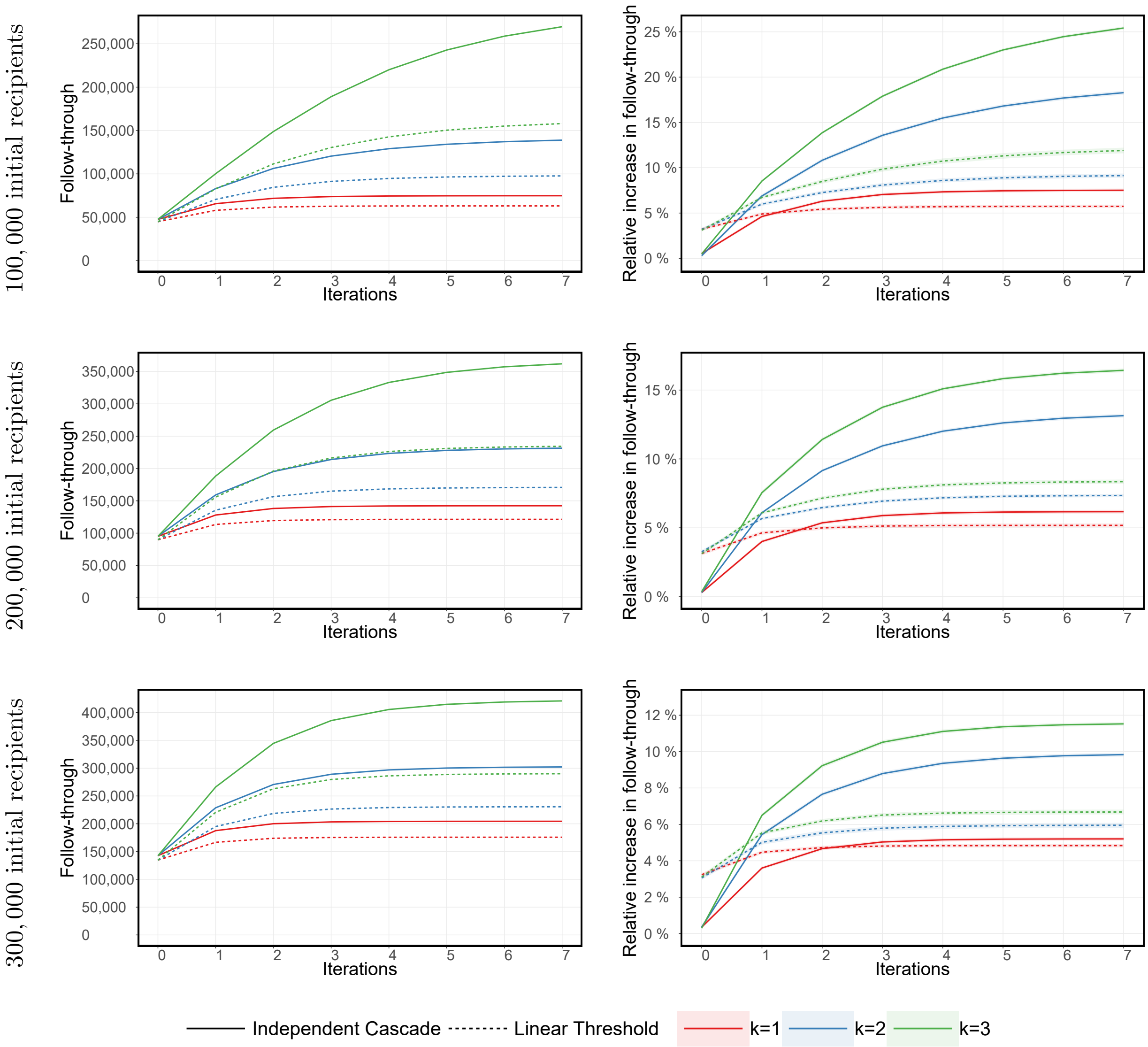

Figure S10: The same as Fig. S7, but for **Erdős-Rényi** networks with a **linear** mapping from the participants' stated propensities (on a Likert scale from 0 to 10) to the actual probabilities (in $[0, 1]$) of them following-through or forwarding the notification.

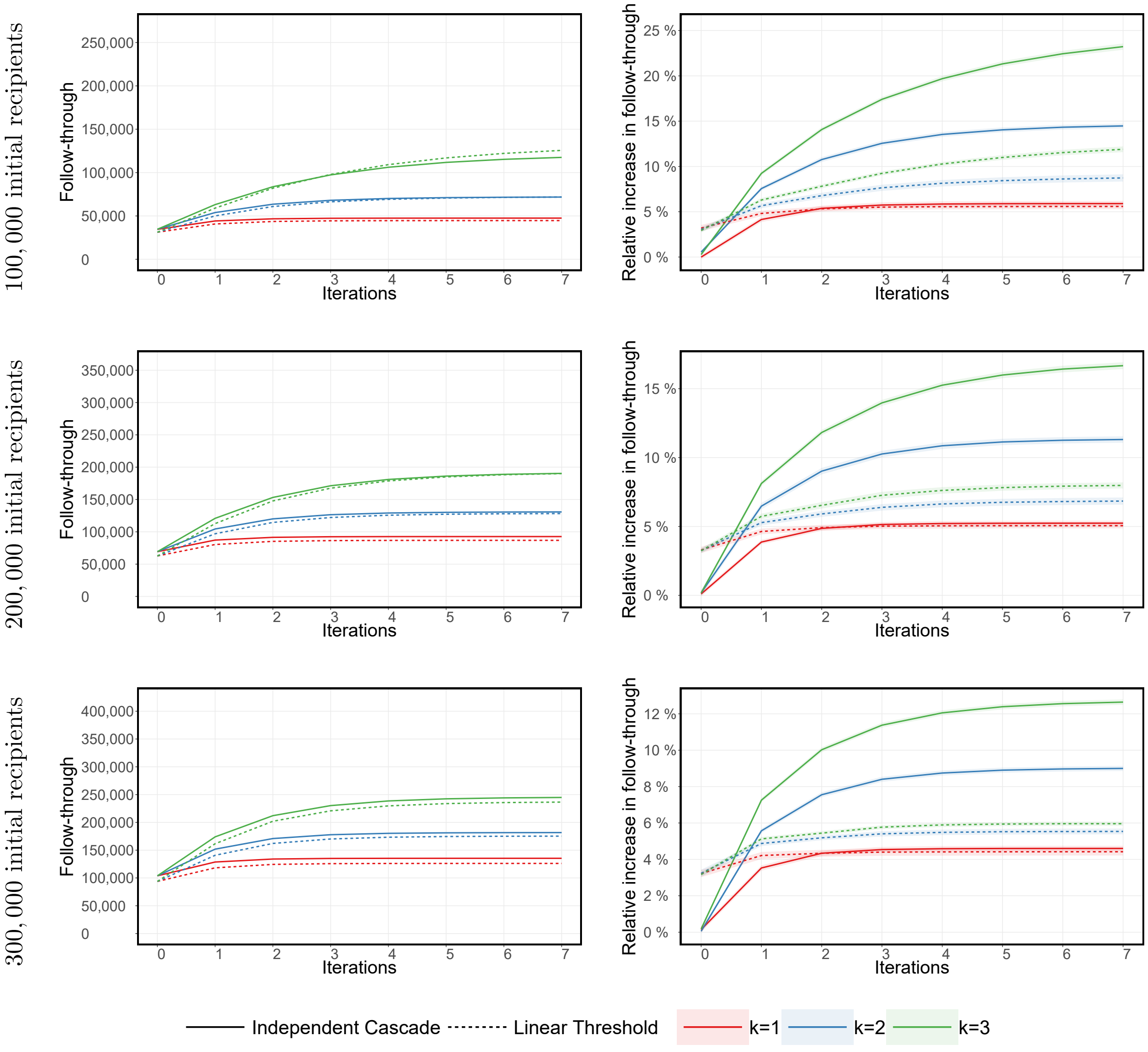

Figure S11: The same as Fig. S7, but for **Erdős-Rényi** networks with a **squared** mapping from the participants' stated propensities (on a Likert scale from 0 to 10) to the actual probabilities (in $[0, 1]$) of them following-through or forwarding the notification.

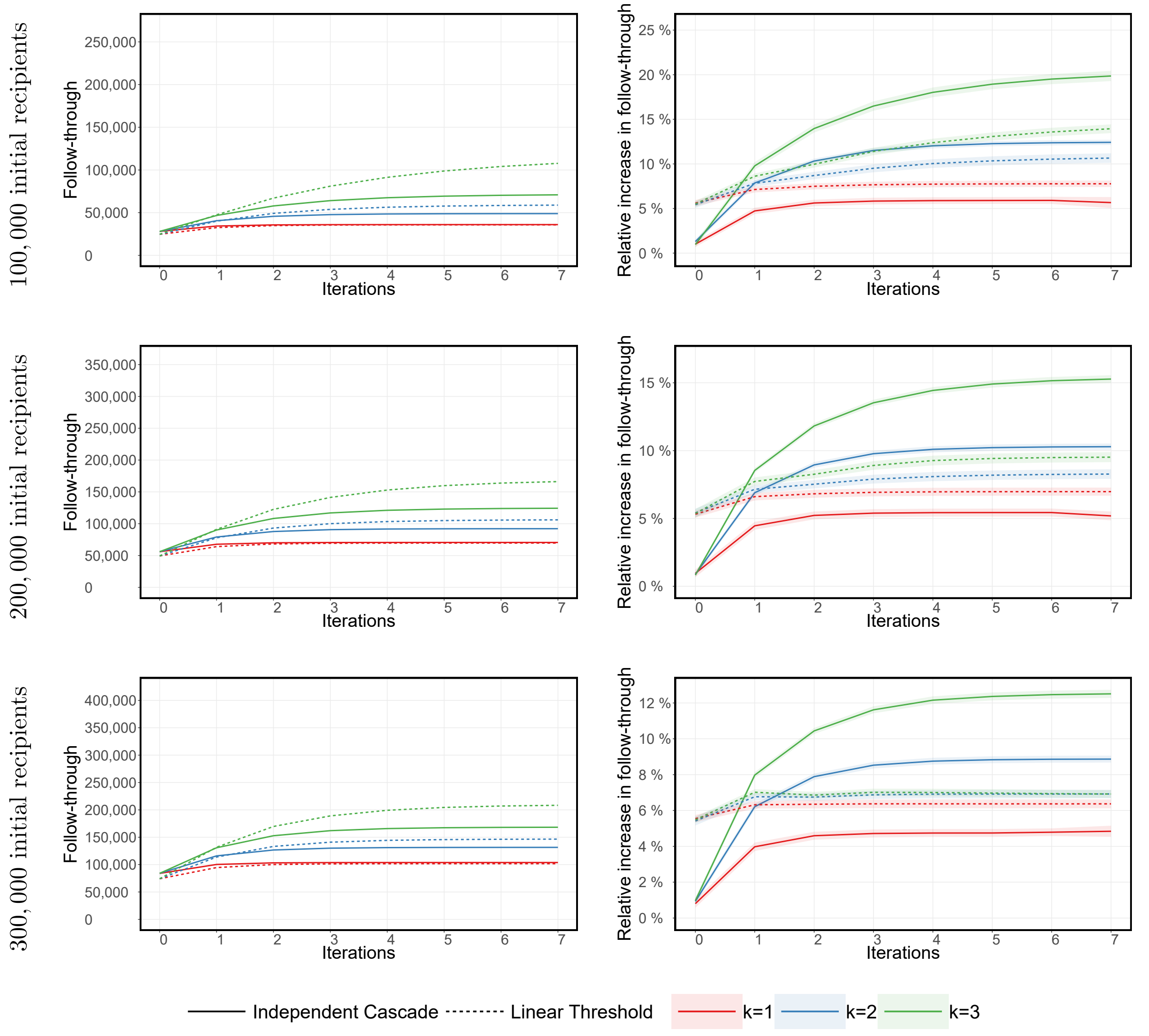

Figure S12: The same as Fig. S7, but for **Erdős-Rényi** networks with a **cubic** mapping from the participants' stated propensities (on a Likert scale from 0 to 10) to the actual probabilities (in $[0, 1]$) of them following-through or forwarding the notification.

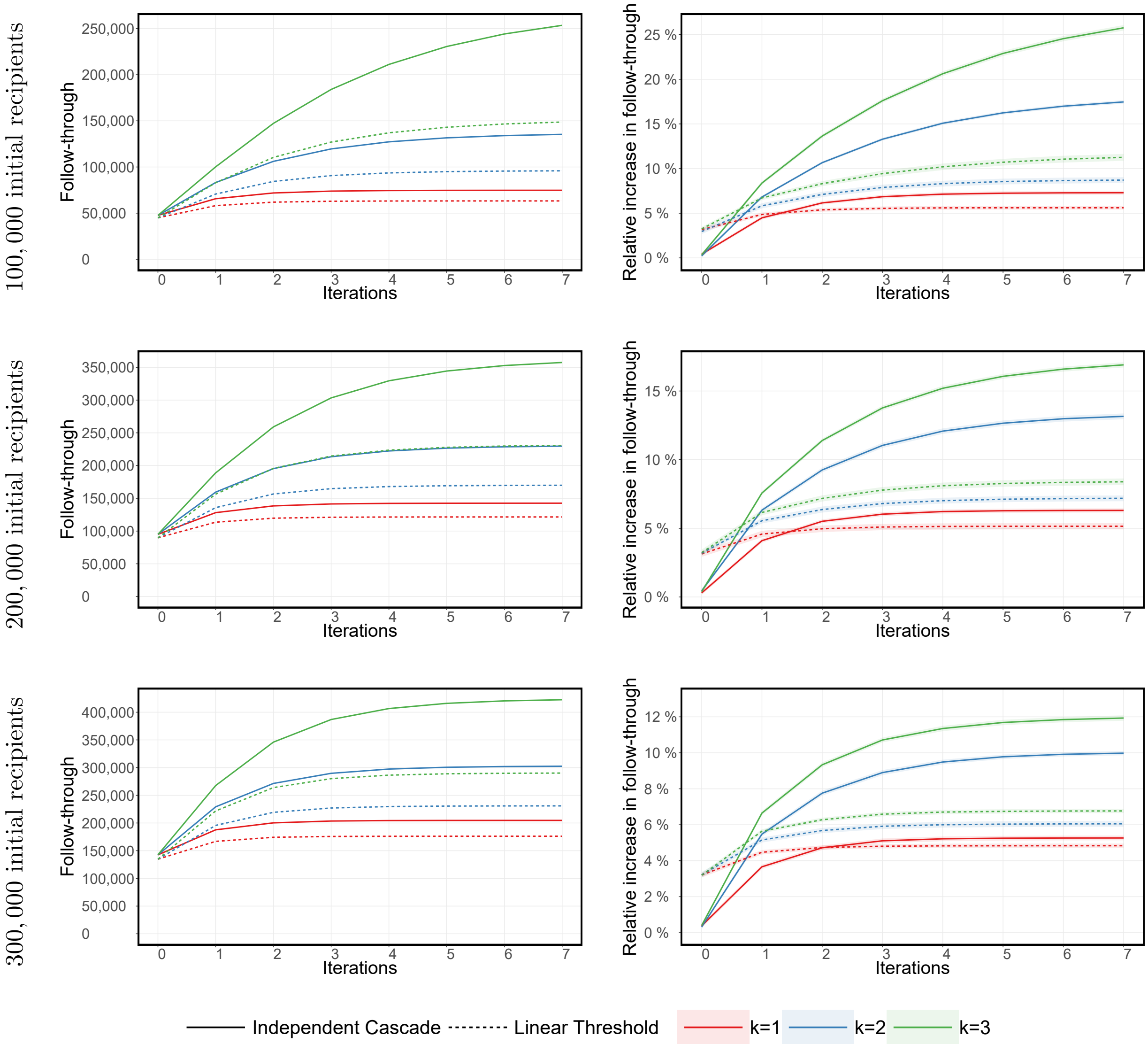

Figure S13: The same as Fig. S7, but for **Watts-Strogatz** networks with a **linear** mapping from the participants' stated propensities (on a Likert scale from 0 to 10) to the actual probabilities (in $[0, 1]$) of them following-through or forwarding the notification.

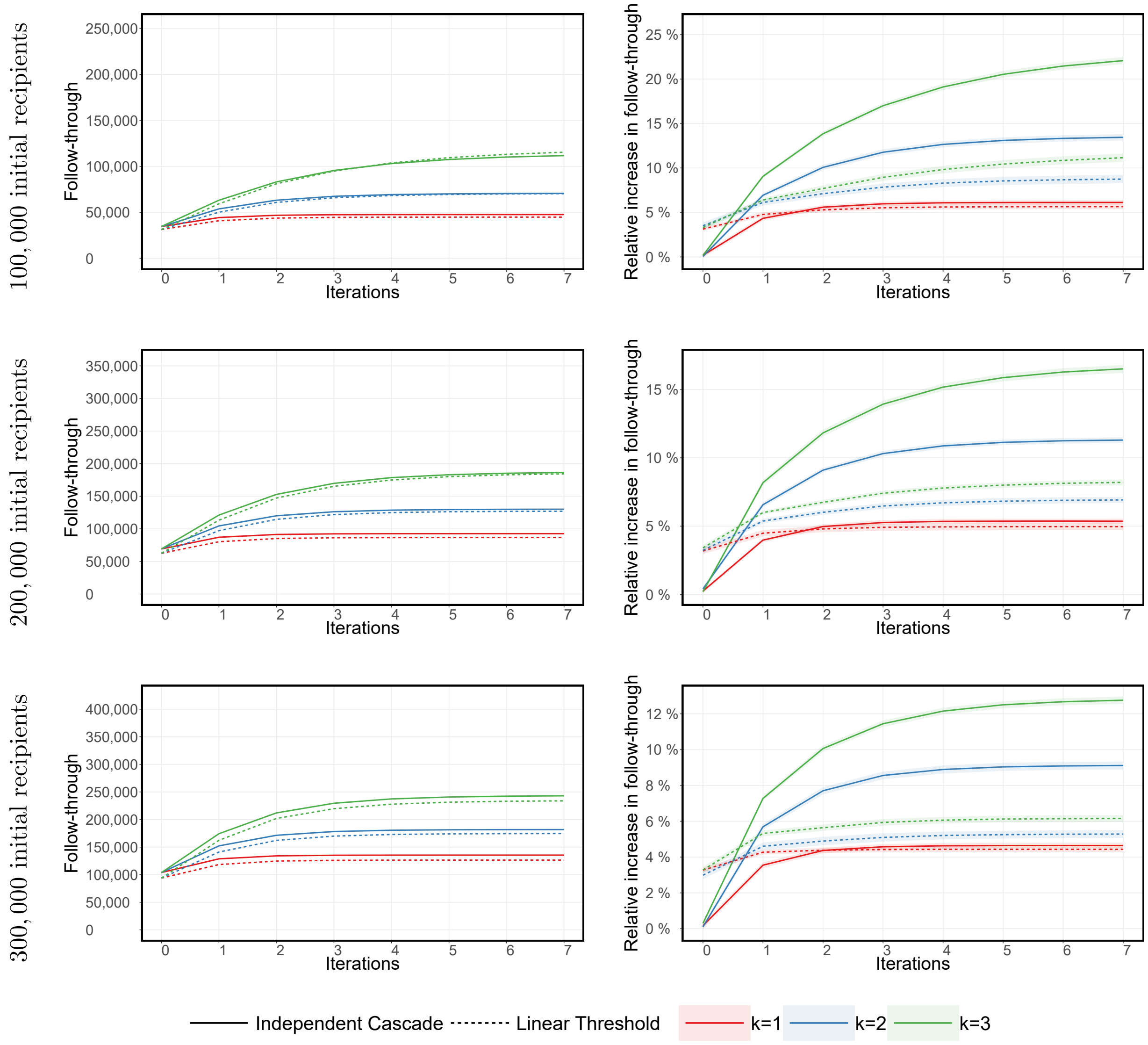

Figure S14: The same as Fig. S7, but for **Watts-Strogatz** networks with a **squared** mapping from the participants' stated propensities (on a Likert scale from 0 to 10) to the actual probabilities (in $[0, 1]$) of them following-through or forwarding the notification.

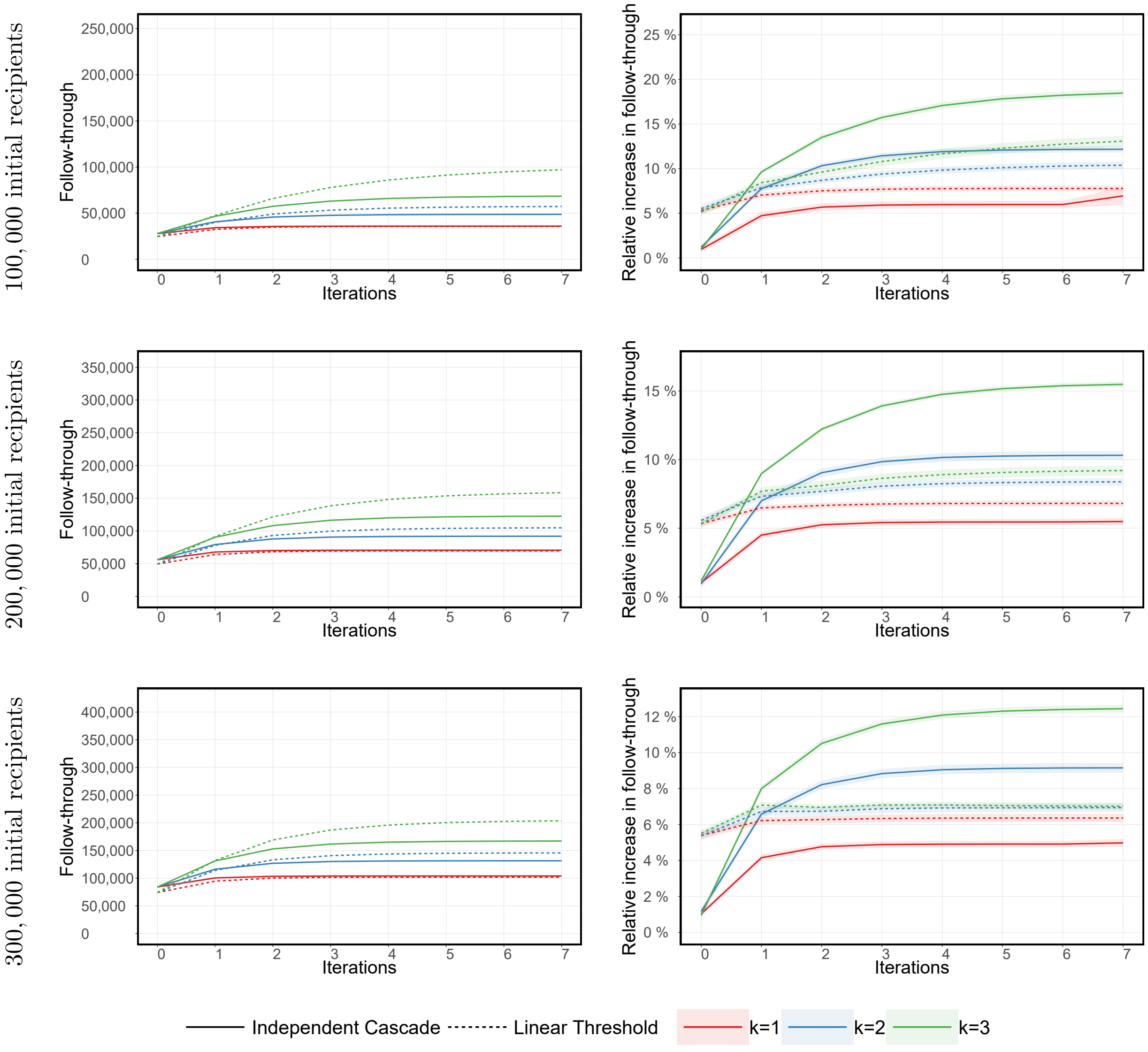

Figure S15: The same as Fig. S7, but for **Watts-Strogatz** networks with a **cubic** mapping from the participants' stated propensities (on a Likert scale from 0 to 10) to the actual probabilities (in $[0, 1]$) of them following-through or forwarding the notification.

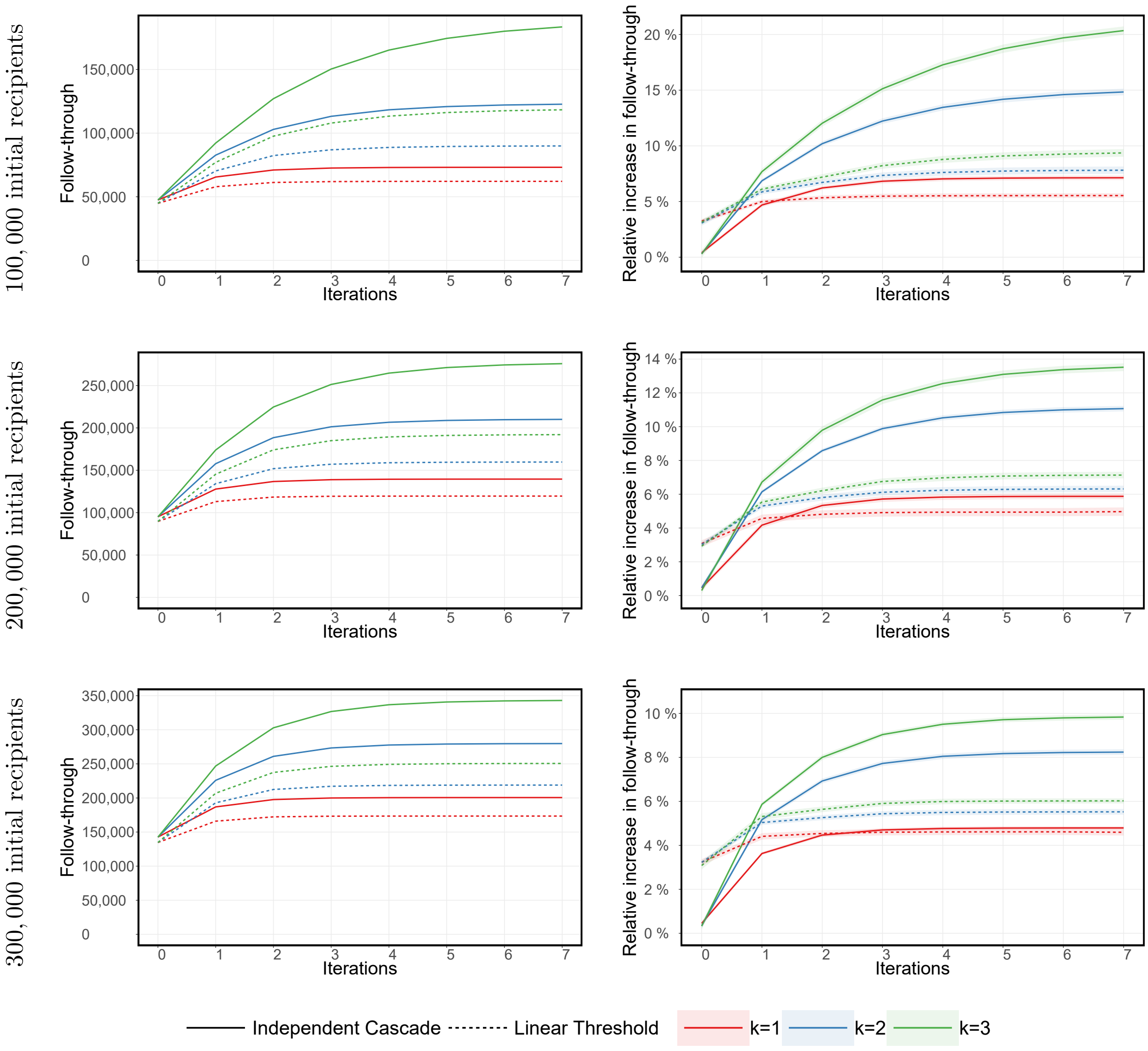

Figure S16: The same as Fig. S7, but for **Newman Configuration** networks with a **linear** mapping from the participants' stated propensities (on a Likert scale from 0 to 10) to the actual probabilities (in $[0, 1]$) of them following-through or forwarding the notification.

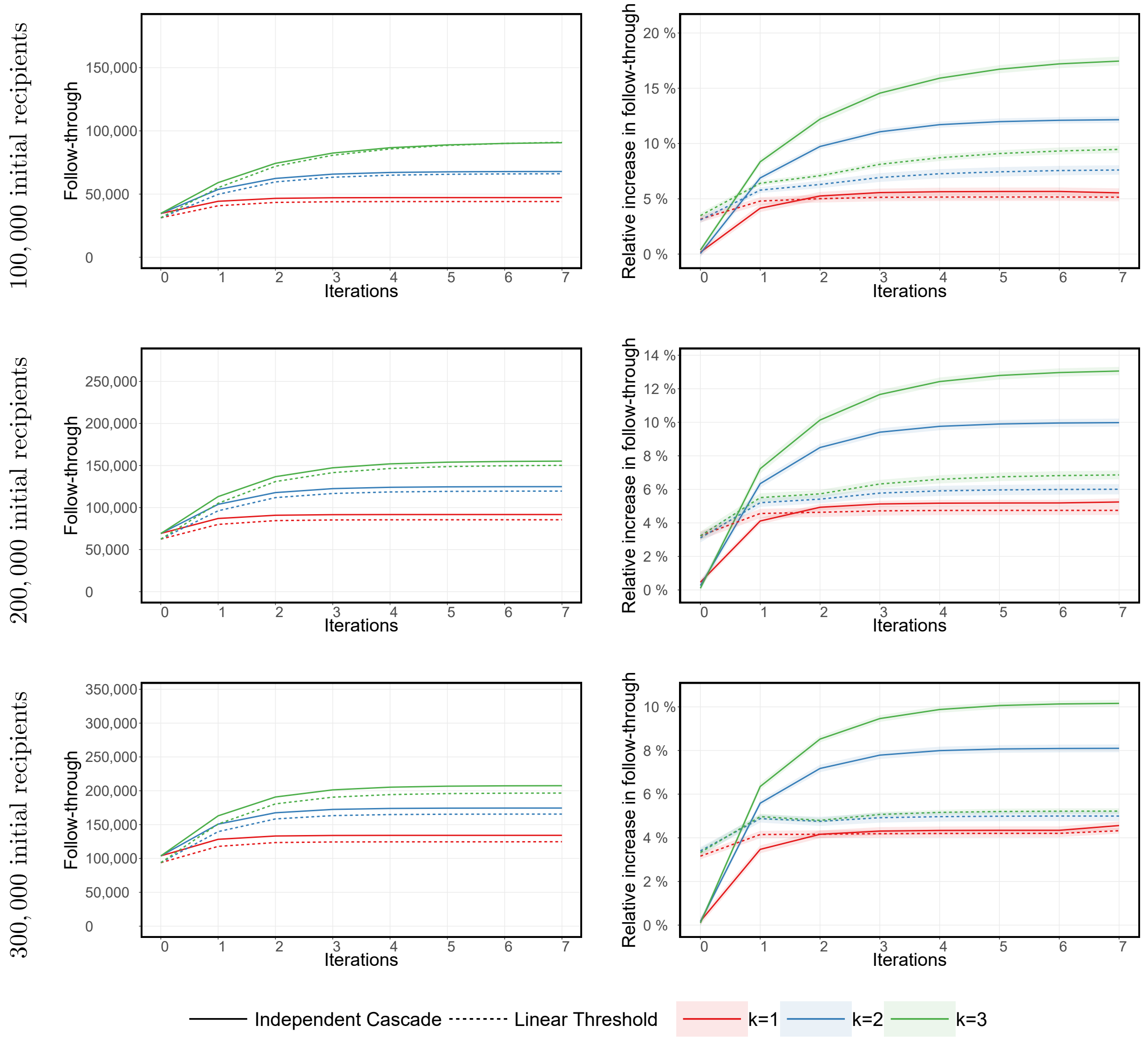

Figure S17: The same as Fig. S7, but for **Newman Configuration** networks with a **squared** mapping from the participants' stated propensities (on a Likert scale from 0 to 10) to the actual probabilities (in $[0, 1]$) of them following-through or forwarding the notification.

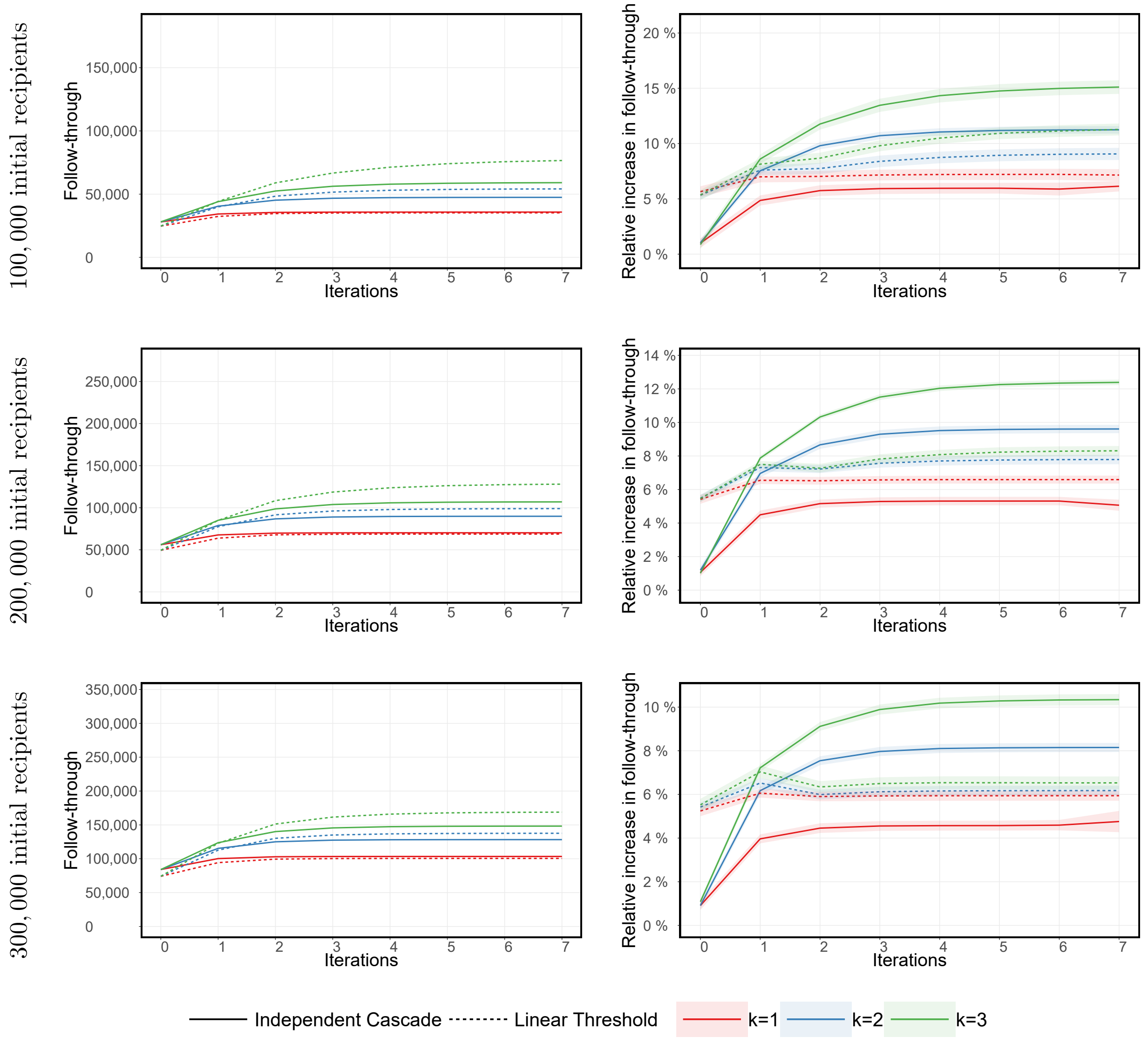

Figure S18: The same as Fig. S7, but for **Newman Configuration** networks with a **cubic** mapping from the participants' stated propensities (on a Likert scale from 0 to 10) to the actual probabilities (in $[0, 1]$) of them following-through or forwarding the notification.

S5 Supplementary note 5: Load profile generation

This section describes how the daily load profile of each residence was generated depending on whether the residents follow-through on the notification and whether they own an electric vehicle (EV).

We first consider the appliances in the residence. The list of appliances depends on the total occupancy of the residence. Each appliance has a probability of being turned on, the value of which varies with the time of the day. For our simulations, we use the appliance list and probability values reported in [10] that describe the energy consumption behavior of a typical UK home. Given this data and the algorithm presented in [10], we generate the residential load profile when no notification is received by the residents. Next, we describe how the load profile changes as a result of the residents following-through on the notification. When the residents follow-through, they shift the use of their deferrable appliances such as the washing machine, dryer, and dishwasher into the peak demand period. Modeling the residents' response as a change in the probability of usage of these appliances would enable us to use the same procedure described in [10] to generate the load profile when the residents do follow-through. To this end, we modify the probability of use of each deferrable appliance, i, at a time, t, denoted by $P_{t,i}$, as follows:

$$P^*_{t,i} = P_{t,i}\ (1-\theta),\ \forall\ t \notin \text{peak period, and} \tag{1}$$

$$P^*_{t_r,i} = P_{t_r,i} + \sum_{\substack{t \notin peak \\ period}} (P_{t,i} - P^*_{t,i}),\ t_r \in \text{peak period.} \tag{2}$$

The variables with asterisks represent the modified values of appliance-use probabilities due to the notification follow-through. Notice that the deferred appliances are rescheduled by the residents to a time t_r chosen randomly within the peak demand period. Here, θ represents the follow-through status of the residence: $\theta = 1$ if the residents follow-through, and $\theta = 0$ if they do not. Fig. S19(a) illustrates the average appliance load profiles obtained for different occupancy values for the two cases when the residents follow-through and when they do not.

A similar procedure is followed to obtain the load profile of the EV if it is present in the residence. The study in [11, 12] presents an algorithm for generating EV profiles based on the observed charging patterns of more than 200 real residential EV users in the UK. Specifically, this algorithm uses the probability that a user begins charging their EV at various times of the day. While using this algorithm in our study to generate the EV load profile when the resident does not follow-through on the notification, we also modify the charging probabilities in order to generate the EV load profiles when the residents do follow-through. In our simulations, the probability of charging the jth EV at a time t, $P^*_{t,j}$, is modified according to the follow-through status, θ, as follows:

$$P^*_{t,j} = P_{t,j}\ (1-\theta),\ \forall\ t \notin \text{peak period, and} \tag{3}$$

$$P^*_{t_r,j} = P_{t_r,j} + \sum_{\substack{t \notin peak \\ period}} (P_{t,j} - P^*_{t,j}),\ t_r \in \text{peak period.} \tag{4}$$

As with the home appliances, the resident, if following-through, schedules the EV to be charged at a time t_r randomly chosen in the peak demand period. Fig. S19(b) illustrates the average load profile obtained for an EV for the two cases when residents follow-through and when they do not.

(a)

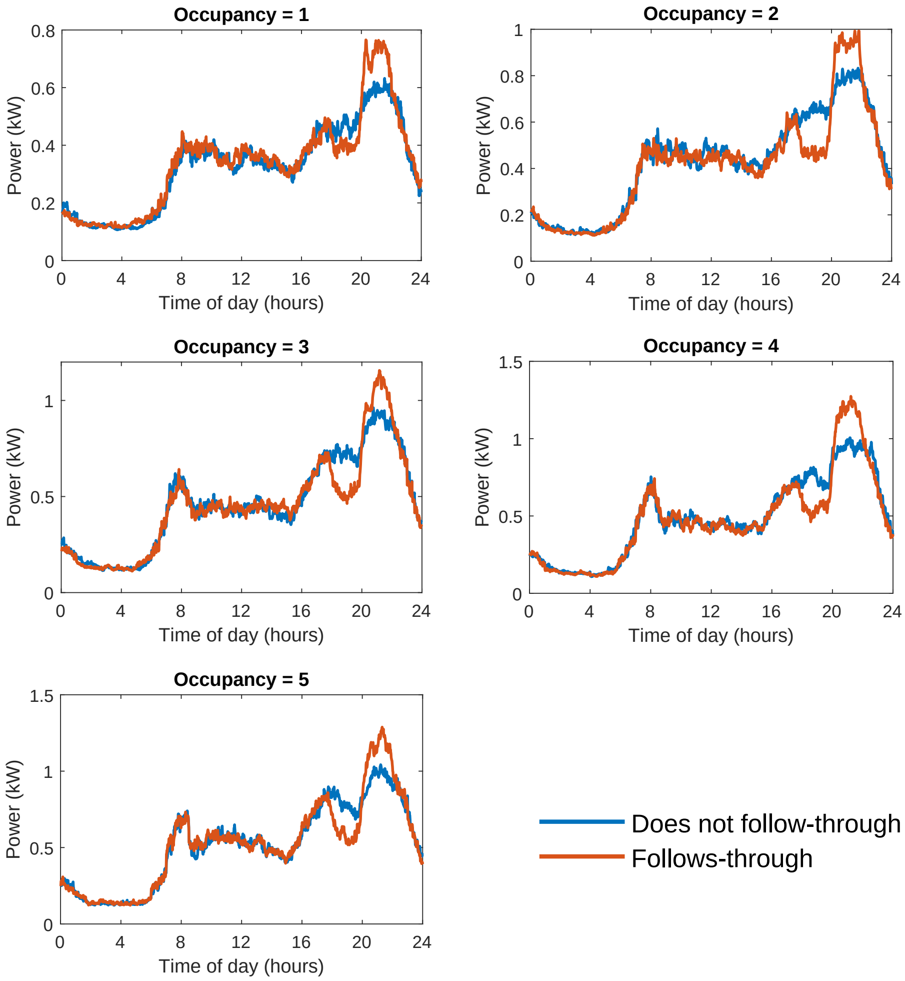

(b)

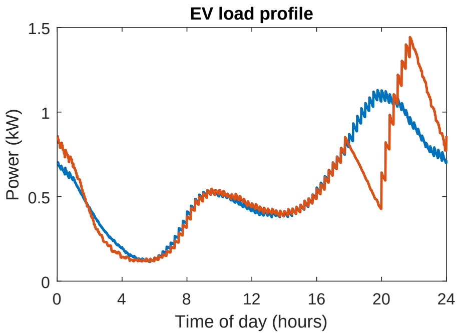

Figure S19: Average daily load profile for (a) appliances in one residence depending on the occupancy, and (b) one electric vehicle. The figure shows load profiles for two scenarios: residents do not follow-through (blue lines), and residents follow-through on the notification (orange lines).

Supplementary References

[1] Open Street Map Wiki. https://wiki.openstreetmap.org/wiki/Key:highway#Values. Accessed: 2019-05-02.

[2] National Grid Electricity System Operator. Appendix c-power flow diagrams, electricity ten year statement 2017. https://www.nationalgrideso.com/document/101736/download.

[3] Joseph B Kruskal. On the shortest spanning subtree of a graph and the traveling salesman problem. *Proceedings of the American Mathematical society*, 7(1):48–50, 1956.

[4] Gururaghav Raman, Bedoor AlShebli, Marcin Waniek, Talal Rahwan, and Jimmy Chih-Hsien Peng. How weaponizing disinformation can bring down a city's power grid: Supplementary data. https://www.penglaboratory.com/topology-data, 2019.

[5] Dale Hall, Hongyang Cui, and Nic Lutsey. Briefing-electric vehicle capitals: Accelerating the global transition to electric drive. *International Council on Clean Transportation*, 2018.

[6] Albert-László Barabási and Réka Albert. Emergence of scaling in random networks. *science*, 286(5439):509–512, 1999.

[7] Paul Erdős and Alfréd Rényi. On random graphs i. *Publ. Math. Debrecen*, 6:290–297, 1959.

[8] Duncan J Watts and Steven H Strogatz. Collective dynamics of small-world networks. *nature*, 393(6684):440–442, 1998.

[9] Mark EJ Newman. The structure and function of complex networks. *SIAM review*, 45(2):167–256, 2003.

[10] Ian Richardson, Murray Thomson, David Infield, and Conor Clifford. Domestic electricity use: A high-resolution energy demand model. *Energy and buildings*, 42(10):1878–1887, 2010.

[11] Jairo Quirós-Tortós, Luis Ochoa, and Timothy Butler. How electric vehicles and the grid work together: Lessons learned from one of the largest electric vehicle trials in the world. *IEEE Power and Energy Magazine*, 16(6):64–76, 2018.

[12] My Electric Avenue. https://www.eatechnology.com/engineering-projects/my-electric-avenue/.
